# Carborane Stabilized “19-Electron” Molybdenum Metalloradical

**DOI:** 10.1021/jacs.1c03568

**Published:** 2021-06-23

**Authors:** Kuldeep Jaiswal, Naveen Malik, Boris Tumanskii, Gabriel Ménard, Roman Dobrovetsky

**Affiliations:** † School of Chemistry, Raymond and Beverly Sackler Faculty of Exact Sciences, Tel Aviv University, Tel Aviv 69978, Israel; ‡ Department of Organic Chemistry, Weizmann Institute of Science, Rehovot 7610001, Israel; § Department of Chemistry and Biochemistry, University of California, Santa Barbara, Santa Barbara, California 93106, United States

## Abstract

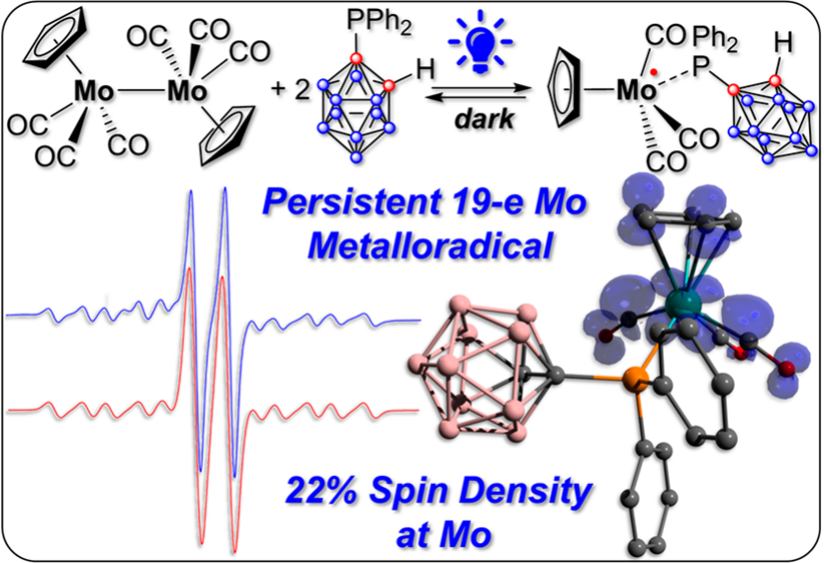

Paramagnetic metal complexes gained a lot of attention due to their participation in a number of important chemical reactions. In most cases, these complexes are dominated by 17-e metalloradicals that are associatively activated with highly reactive paramagnetic 19-e species. Molybdenum paramagnetic complexes are among the most investigated ones. While some examples of persistent 17-e Mo-centered radicals have been reported, in contrast, 19-e Mo-centered radicals are illusive species and as such could rarely be detected. In this work, the photodissociation of the [Cp(CO)_3_Mo]_2_ dimer (**1**) in the presence of phosphines was revisited. As a result, the first persistent, formally 19-e Mo radical with significant electron density on the Mo center (22%), Cp(CO)_3_Mo^**•**^PPh_2_(*o*-C_2_B_10_H_11_) (**5b**), was generated and characterized by EPR spectroscopy and MS as well as studied by DFT calculations. The stabilization of **5b** was likely achieved due to a unique electron-withdrawing effect of the *o*-carboranyl substituent at the phosphorus center.

## Introduction

Organometallic chemistry is mostly dominated by diamagnetic complexes, which obey the 16- and 18-electron rule.^1,2^ This rule is very useful for predicting the stability and reactivity of diamagnetic metal complexes. Since the 1980s, however, paramagnetic metal complexes began to gain significant attention due to their important role in a variety of chemical reactions.^3−7^ For instance, paramagnetic metal intermediates of the second and third rows are involved in redox reactions, chain mechanisms, homolytic cleavage, and catalysis of C–C bond formation^3,8^ as well as in mediating redox reactions in energy-conversion processes and in biomimetic C–H bond activation and epoxidation of hydrocarbons.^9^ Their intermediacy in industrial processes such as the Wacker reaction is also well-known.^10^ As a result, the range of organometallic chemistry has expanded to include numerous paramagnetic 17-e complexes, which exist as both stable complexes and short-lived intermediates.^3,4,8,11,12^ The reactions of paramagnetic 17-e metal complexes are associatively activated with 19-e intermediates or transition states.^3,4,11^ However, unlike 17-e metal complexes, 19-e metal complexes in which the unpaired electron is primarily metal localized in a M–L antibonding orbital are rare and usually unstable and as such were proposed mostly as illusive intermediates.^4,12,13^ The 19-e Mo-centered radicals, to the best of our knowledge, have never been observed in chemical reactions, with the exception of femtosecond IR spectroscopy.^14,15^ Noteworthy, persistent 19-e Mo-centered radicals that are perhaps better described as 18-e complexes with reduced ligands (so-called “18 + δ” complexes) were synthesized previously; however, the spin density on the metal center in these complexes was negligible (<1%).^16−19^

**Scheme 1:**
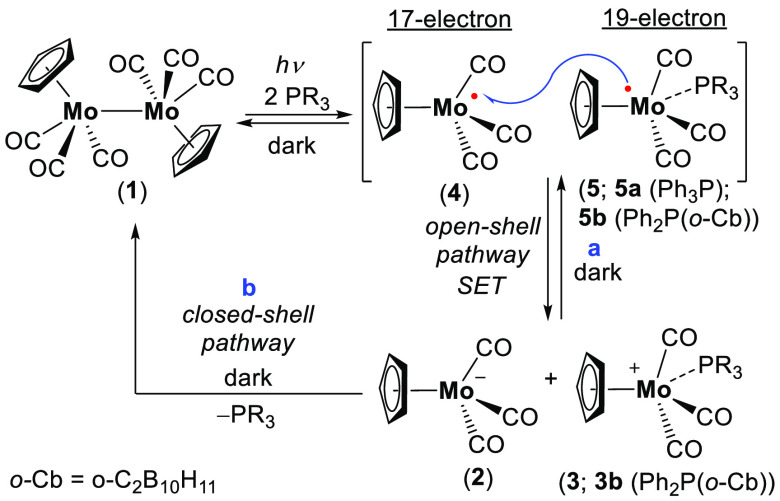
Mechanism of [Cp(CO)_3_Mo]_2_ Dimer (**1**) Dissociation in the Presence of R_3_P; Formation of Cp(CO)_3_(R_3_P)Mo^+^ (**3**) and Cp(CO)_3_Mo^–^ (**2**)

One of the earliest reactions postulated to involve a 19-e intermediate was the photochemical disproportionation of the [Cp(CO)_3_Mo]_2_ dimer (**1**) in the presence of R_3_P into the Cp(CO)_3_Mo^–^ (**2**) and Cp(CO)_3_(R_3_P)Mo^+^ (**3**) ion pair (Scheme 1).^13−15^ The accepted mechanism for this reaction, proposed by Tyler and co-workers,^13^ proceeds through photoexcitation of **1** leading to Mo–Mo bond cleavage and the formation of two 17-e Cp(CO)_3_Mo^**•**^ radicals (**4**). In the presence of R_3_P the formation of a highly reducing, transient 19-e intermediate Cp(CO)_3_(R_3_P)Mo^**•**^ (**5**) is proposed. Electron transfer from **5** to **4** leads to formation of Cp(CO)_3_(R_3_P)Mo^+^ (**3**) and Cp(CO)_3_Mo^–^ (**2**) (Scheme 1). Importantly, it was also shown that this reaction is reversible, and in the dark, this salt over time is converted back to **1** and R_3_P, either via a single electron transfer (SET) path (Scheme 1a) or directly by substitution of R_3_P (Scheme 1b) with no clear indication as to which mechanism prevails in this transformation.^13,20^

Noteworthy, when **1** was irradiated in the presence of a bidentate diphosphine-based ligand (2,3-bis(diphenylphosphino)maleic anhydride)), a stable isolable 18 + δ complex was formed with most of the spin density located at the diphosphine ligand (i.e., δ was close to zero).^16−18^ On the other hand, when R_3_P in this reaction (Scheme 1) was replaced by an *N*-heterocyclic carbene (NHC), a persistent 17-e Cp(CO)_2_(NHC)Mo^**•**^ radical was formed via substitution of one of the COs by the carbene.^21^ Noteworthy, neither 17-e **4** nor 19-e **5** was observed or characterized by electron paramagnetic resonance (EPR) spectroscopy.

We, therefore, decided to revisit this reaction (Scheme 1) and see whether radicals of type **5** could be stabilized in this process and studied by EPR spectroscopy. Herein, we report the generation and characterization of the *first persistent formally 19-e Mo-based radical with a significant spin density of 22% on the Mo center*, Cp(CO)_3_Mo^**•**^PPh_2_(*o*-C_2_B_10_H_11_) (**5b**).

## Results and Discussion

**Figure 1:**
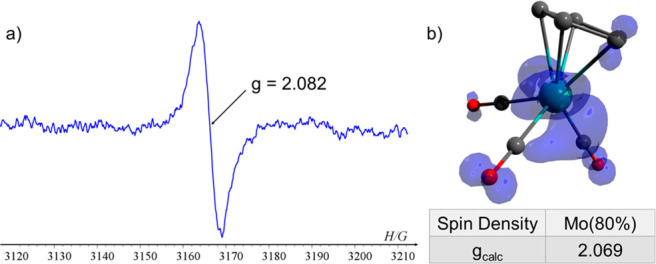
(a) EPR spectrum of **4**. (b) DFT calculated Mulliken atomic spin densities and *g*-value of **4**.^23^

**Scheme 2:**
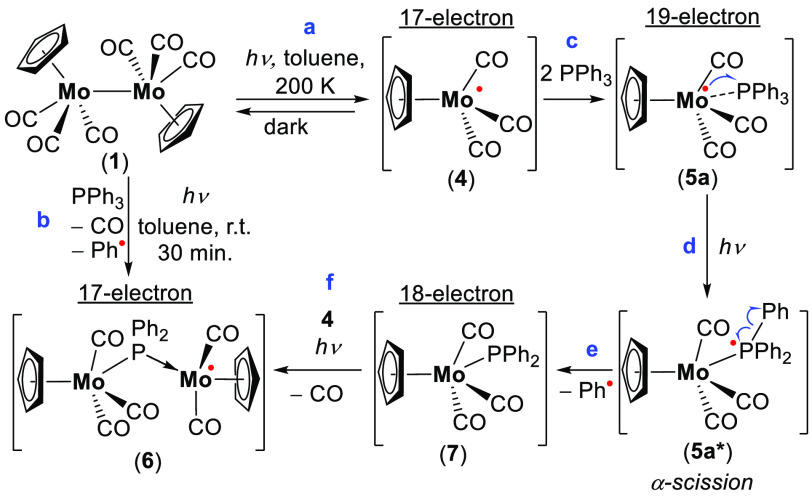
Photochemical Reaction of Dimer **1** in the Presence of Excess of Ph_3_P and Formation of an Unstable Cp(CO)_3_Mo^•^ (**4**) and a Persistent **6**, with Proposed Pathway Leading to **6**

We first studied the photochemical reaction of **1** in the presence of Ph_3_P. Thus, when a toluene solution^22^ containing **1** and Ph_3_P (1:10) in the EPR cavity was UV-irradiated (λ > 300 nm) at low temperature (200 K), a strongly low-field shifted singlet with a *g*-value of 2.082 was measured (Figure 1a), which immediately disappeared when irradiation was stopped. We assumed that this signal corresponded to the transient 17-e Cp(CO)_3_Mo^**•**^ radical (**4**) (Scheme 2a). To support our suggestion, **4** was optimized by using DFT (density functional theory), and its EPR parameters were calculated.^23^ The calculated *g*-value of **4** (*g* = 2.069) is in good agreement with the experimentally observed *g*-value (Figure 1b). To the best of our knowledge, this is the first time that the “parent” 17-e Cp(CO)_3_Mo^**•**^ radical (**4**) was experimentally observed by EPR spectroscopy.

**Figure 2:**
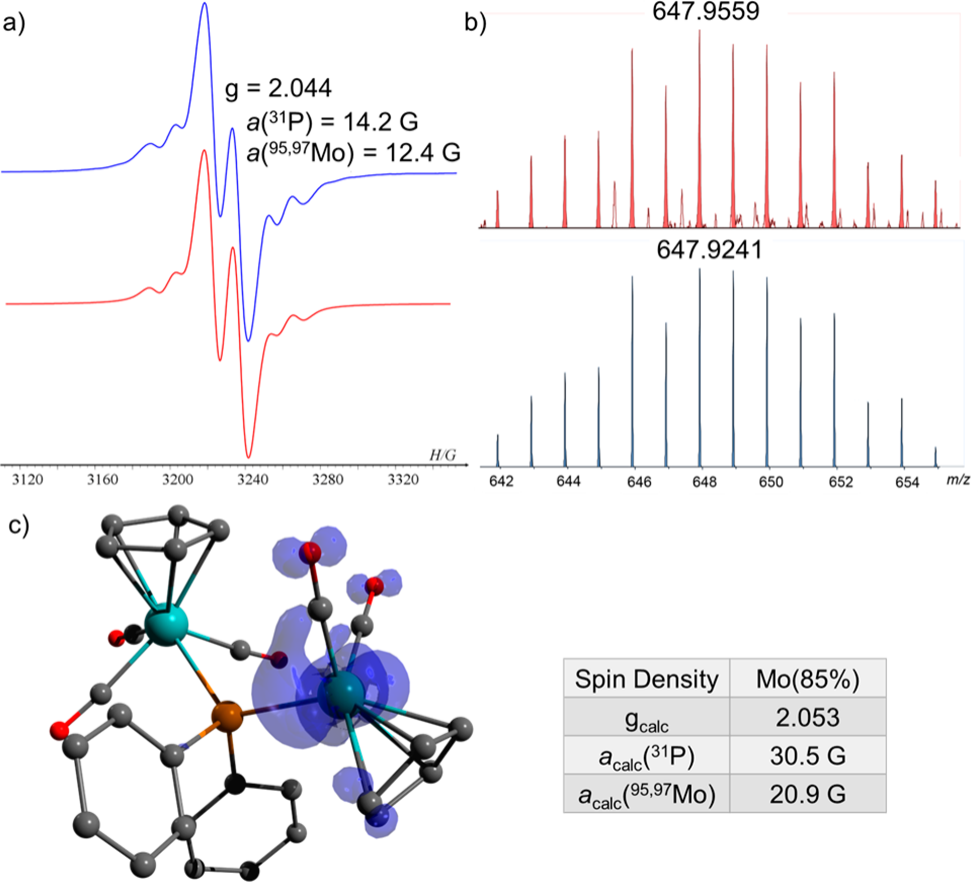
(a) EPR spectrum of **6** (blue) and its simulation (red). (b) MS of **6** (647.9559 (M + H)^+^) (red) and its simulation (blue). (c) DFT calculated Mulliken atomic spin densities and EPR parameters in **6**.^23^

After irradiation (λ > 300 nm) of the same toluene solution (**1** and Ph_3_P (1:10)) at room temperature for 30 min, a high-intensity doublet was measured (14.2 G) with a *g*-value of 2.044 and a hyperfine coupling *a*(^95,97^Mo) = 12.4 G from magnetically active Mo isotopes (Figure 2a). This radical species was persistent with τ_1/2_ ≈ 180 min and thus allowed us to study its molecular composition using mass spectrometry (MS). Using atmospheric pressure chemical ionization (APCI) MS in positive mode, we were able to detect a mass that corresponds to a 17-e Mo-centered radical (647.9559 (M + H)^+^) (Figure 2b), Cp(CO)_2_Mo^**•**^ ← P(Ph)_2_–Mo(CO)_3_Cp (**6**) (Scheme 2b).

DFT calculation of **6** and its EPR parameters^23^ gave a *g*-value of 2.053 with hyperfine coupling constants (hfcc) of *a*(^31^P) = 30.54 G, *a*(^95^,^97^ Mo) = 20.96, and spin density located mostly on the Mo atom (85%) (Figure 2c). The computed EPR parameters are in good agreement with the experimental values considering the low spin density at the phosphorus center and rather complicated electronic structure of the Mo atom.^24,25^

We assumed that **6** was formed via unstable 19-e Mo-based intermediate **5a** under irradiation (Scheme 2c). The unpaired electron in **5a** can migrate to the σ* orbital at the phosphorus center under irradiation, giving the excited species 5a* (Scheme 2d).^23,26^5a* resembles in its electronic structure phosphoranyl radicals (R_4_P^•^), which tend to decay via α- or β-scission reactions,^27−29^ and thus 5a* decays in a similar manner via α-scission of the Ph–P bond, giving 18-e Cp(CO)_3_MoPPh_2_ (**7**) (Scheme 2e).^30^**7** may then substitute one of the CO groups at **4** to give **6** (Scheme 2f). A similar type of photoinduced CO substitution was previously reported.^13,21^

**Scheme 3:**
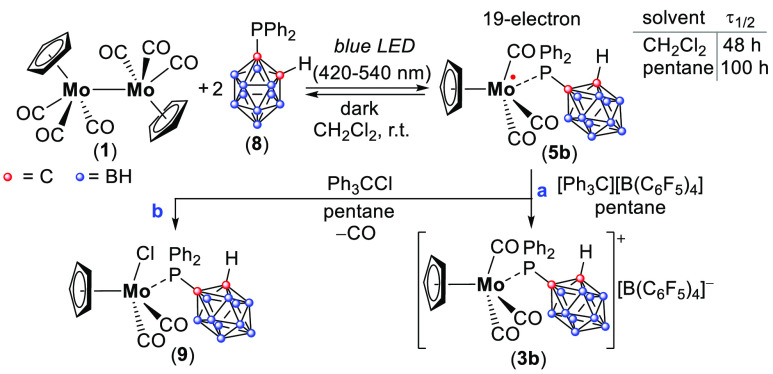
Reaction of Dimer **1** with Ph_2_P(*o*-C_2_B_10_H_11_) (**8**) to Generate the Persistent Radical **5b**; Decay of **5b** by Cl Atom Abstraction Giving **9** (b) and Oxidation of **5b** by [Ph_3_C][B(C_6_F_5_)_4_] Giving **3b** (a)

To overcome the problem of instability of **5a**, especially under irradiation (see Scheme 2d,e), we decided to replace Ph_3_P by Ph_2_P(*o*-C_2_B_10_H_11_) (**8**) (Scheme 3).^31,32^ We envisioned that this substitution will solve a few of the problems that we encountered when using Ph_3_P (Scheme 2). First, Ph_2_P(*o*-C_2_B_10_H_11_) (**8**) is a weaker donor due to the strong electron-withdrawing effect of the *o*-carboranyl group^33−39^ and thus will lead to a less electron-rich Mo center, which would make the desired 19-e complex less reducing and as a result more stable.^40^ Second, the *o*-carboranyl substituent at the phosphorus center could help overcome the instability of **5a** under irradiation. In contrast to **5a**, which under irradiation is excited to a phosphoranyl-type radical 5a*, which decays via α-scission reaction (see Scheme 2d,e), in **5b** the photoinduced electron migration would most probably lead to the migration of the spin density into the *o*-carboranyl cage, an effect that was previously shown by our and other groups.^41−45^ This may prevent the decay of **5b** radical by α-scission (Scheme 2e).

**Figure 3:**
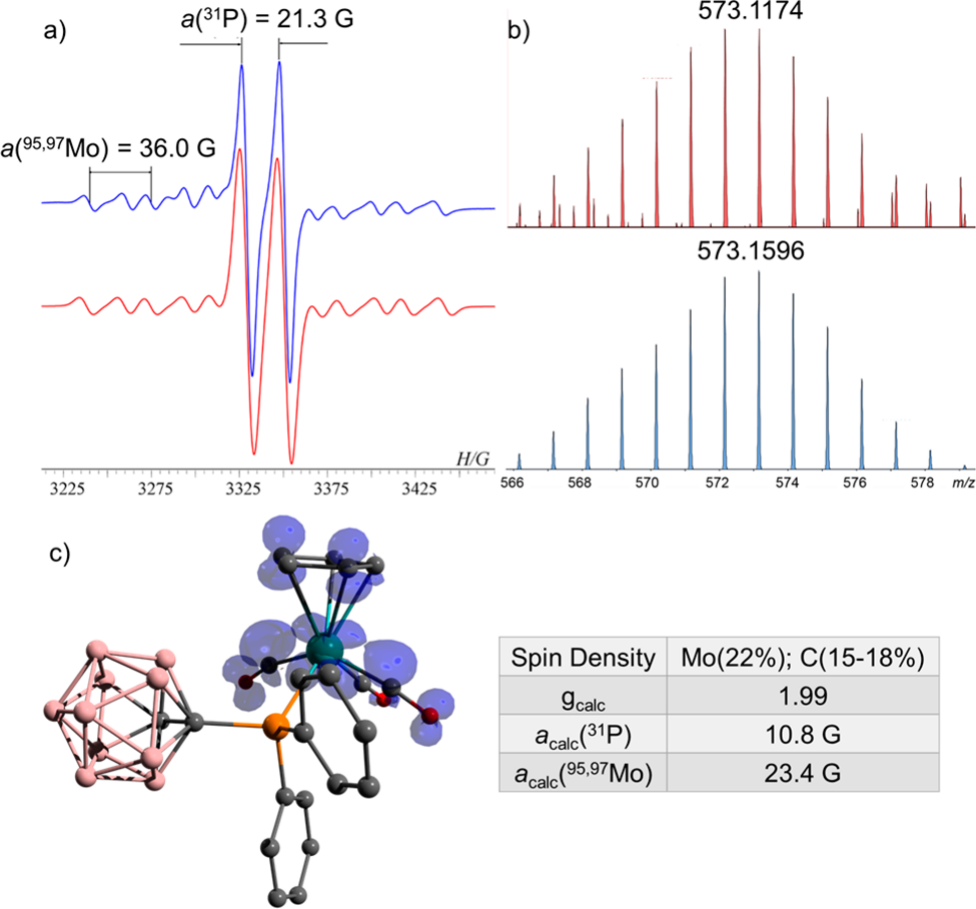
(a) EPR spectrum of **5b** (blue) and its simulation (red). (b) MS of **5b** (573.1174 (M–H)^−^) (red) and its simulation (blue). (c) DFT calculated Mulliken atomic spin densities and EPR parameters in **5b**.^23^

The reaction between **1** and **8** (1:10) in toluene under UV irradiation (λ > 300 nm, 30 min) did not produce the desired radicals. However, when the solvent was changed to CH_2_Cl_2_,^22^ and the solution of **1** and **8** (1:2) was irradiated with visible light from a 34 W blue LED lamp (λ = 420–540 nm) for 1 h,^46^ the desired radical **5b** was generated (Scheme 3) and was stable enough to study by EPR spectroscopy and MS methods (Figure 3).

The EPR spectrum of **5b** (*g* = 1.980) is characterized by the hfcc with the ^31^P nucleus *a*(^31^P) = 21.3 G and magnetically active Mo isotopes *a*(^95,97^Mo) = 36.0 G (Figure 3a). The geometry of **5b** was DFT optimized, and its EPR parameters were calculated.^23^ The calculated *g*-value (1.990) is in good agreement with the experimental *g*-value (1.980), with an hfcc of *a*(^31^P) = 10.8 G and *a*(^95,97^Mo) = 23.4 G. The spin density is distributed between Mo (22%) and carbon atoms of the CO and Cp substituents (15–18%, for each carbon) (Figure 3c).^23^ Noteworthy, the spin density on the phosphine ligand (**8**) is negligible (2.4%).^23^ The mass corresponding to radical **5b** (573.1174 (M–H)^−^) was found in the MS of the reaction mixture by using APCI MS in negative mode (Figure 3b). Noteworthy in **5b**, the lower spin density on the Mo center (22%), as well as the negatively shifted *g*-value compared to a free electron (Δ*g* = −0.0223), clearly contrasts with the higher spin density and positively shifted Δ*g* of the 17-e Mo-centered radicals **2** (80%, Δ*g* = 0.0797) and **6** (85%, Δ*g* = 0.0417) (Schemes 1 and 2).

To the best of our knowledge, this is the first time that a persistent formally 19-e Mo-based radical complex with significant electron density on Mo (22%) was generated and studied spectroscopically. In contrast, doing the reaction between **1** and Ph_3_P (1:2) under the same reaction conditions did not yield the 19-e Mo-based radical **5a**, but radical **6** was observed by EPR spectroscopy (Scheme 2), meaning that the *o*-carboranyl substituent at the P center indeed plays a crucial role in stabilizing this type of radical.

Expectedly, in the dark, **5b** was not stable over long periods of time (τ_1/2_ ≈ 48 h), and after a few days only the starting materials, **1** and **8**, were detectable by NMR spectroscopy. Notably, when the same reaction mixture was irradiated again (λ = 420–540 nm), **5b** was regenerated. Similar to the described reaction in Scheme 1, we assume that **5b** is a persistent 19-e Mo radical intermediate of the dissociation reaction of **1** in the presence of **8**.^13,20^ Interestingly, **5b** extracted by pentane is more stable than in CH_2_Cl_2_ solution with τ_1/2_ ≈ 100 h.

**Figure 4:**
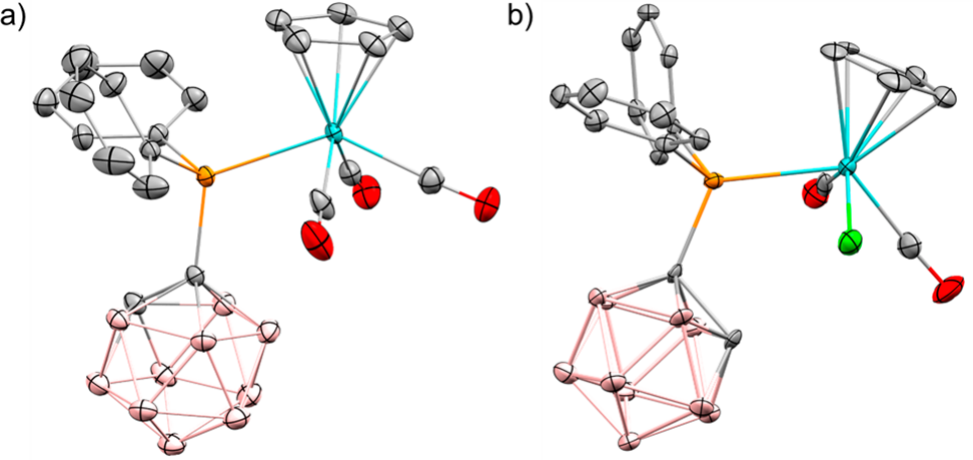
POV-ray depiction of cation **3b** (a) and of **9** (b); thermal ellipsoids at the 50% probability level. Hydrogens and the [B(C_6_F_5_)_4_]^−^ anion are omitted for clarity.

Oxidation of **5b** was achieved by its reaction with [Ph_3_C][B(C_6_F_5_)_4_], giving the corresponding cation [Cp(CO)_3_(Ph_2_(*o*-C_2_B_10_H_11_)P)Mo]^+^ (**3b**) (Scheme 3a), which was also independently synthesized, isolated, and fully characterized (X-ray molecular structure shown in Figure 4a).^47^ Noteworthy, **3b** was also observed by ^31^P NMR (see Figure S20) in the photodissociation process as consequent reaction of radicals **5b** and **4**, similarly to the reaction shown in Scheme 1.

Reaction of **5b** with Ph_3_CCl in pentane produced Cp(Cl)(CO)_2_MoPPh_2_(*o*-C_2_B_10_H_11_) (**9**) (Scheme 3b), the product of Cl atom abstraction and decarbonylation (for the EPR spectrum of this reaction see Figure S22).^45^**9** was isolated by crystallization, and its molecular structure was determined by X-ray crystallography (Figure 4b).

**Figure 5:**
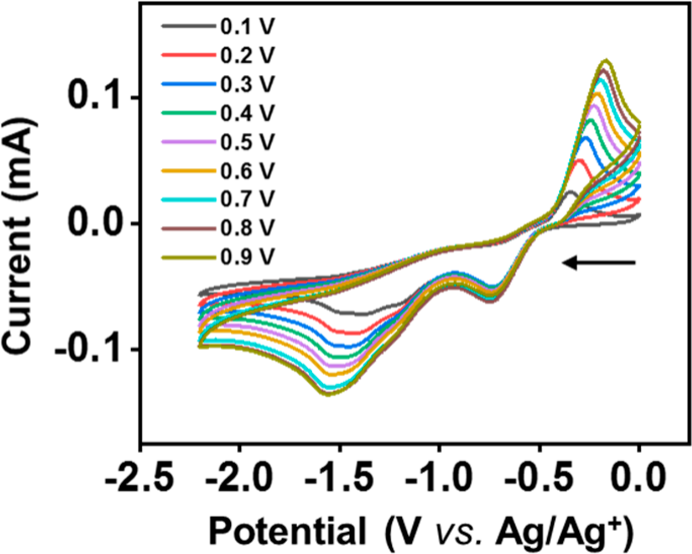
CV of **3b** (5.99 mM) in dry 0.1 M [*n*Bu_4_N][B(C_6_F_5_)_4_]/CH_2_Cl_2_ solution obtained at various scan rates with glassy carbon electrodes, Pt wire, and Ag/Ag^+^ as the working, counter, and reference electrodes, respectively.

The redox chemistry of **3b** was studied by both cyclic voltammetry (CV) and chemical reduction experiments. The CV of **3b** in CH_2_Cl_2_ (5.99 mM) using [nBu_4_N][B(C_6_F_5_)_4_] (0.1 M) as a supporting electrolyte was collected and revealed irreversible reduction events centered at = −0.736 V and = −1.377 V with a corresponding anodic event at = −0.341 V vs the Ag/Ag^+^ redox couple at a scan rate of 100 mV/s (Figure 5).^45^ The large peak-to-peak separation (395 mV) between and suggests significant structural reorganization upon reduction of **3b**.

**Scheme 4:**
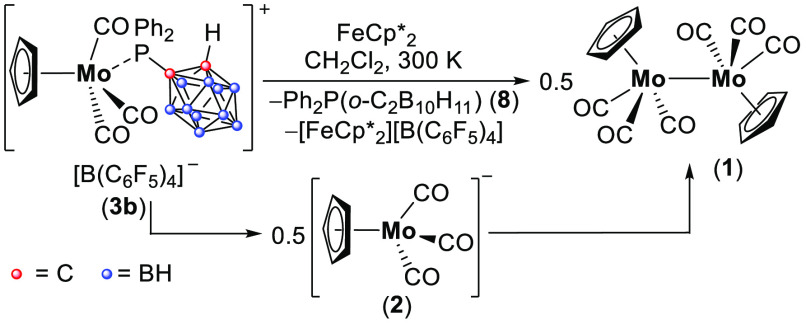
Reduction Reaction of Cation **3b** by FeCp*_2_

On the basis of the CV results, we attempted to isolate the product of the first reduction event by using FeCp*_2_ as a reductant (−1.13 V vs Ag/Ag^+^ in CH_2_Cl_2_). Thus, when **3b** was reacted with 1 equiv of FeCp*_2_ in CH_2_Cl_2_, 0.5 equiv of **3b** was consumed and 0.5 equiv of free phosphine **8** and the anion [Cp(CO)_3_Mo]^−^ (**2**) were produced. Over time, **3b** was consumed totally, leading to **1** and free **8**, likely the result of the reaction of the remaining 0.5 equiv of **3b** with intermediate **2** (Scheme 4).^45^ Noteworthy, we did not observe the formation of **5b** in this process by EPR spectroscopy, suggesting that a rapid 2e reduction process dominates this transformation. This also suggests that the peak in the CV is a 2e event. Importantly, similar 2e reduction processes were reported previously.^4,48,49^ Because no paramagnetic species were observed at all stages of this experiment (Scheme 4), we assume that the reaction of **3b** and **2** most likely proceeds via a closed-shell pathway and not through radicals **5b** and **4** (see Scheme 1b).

## Conclusion

To conclude, in this work the photodissociation of **1** in the presence of Ph_3_P and **8** was performed, and thorough EPR studies were done. The photochemical reaction of **1** with Ph_3_P in toluene led to the formation of the persistent 17-e Mo-centered radical complex **6** via a transient “parent” 17-e complex Cp(CO)_3_Mo^•^ (**4**), which was detected by EPR for the first time. **6** is presumably formed under irradiation which induces α-scission reaction of a P–Ph bond, followed by adduct formation with **4**. To overcome this problem, **8** was used instead of Ph_3_P, which in reaction with **1** in CH_2_Cl_2_ under irradiation at λ = 420–540 nm gave the persistent formally 19-e Mo-based radical **5b**. Accessing what previously had only been a hypothesized intermediate in Mo chemistry allowed us to carry out some preliminary reactivity studies. Oxidation of **5b** by [Ph_3_C][B(C_6_F_5_)_4_] gave the corresponding cation **3b**. The reaction of **5b** with alkyl chlorides gave **9** via Cl atom abstraction and decarbonylation. The electrochemical reduction of **3b** proceeds via two irreversible reduction events. To study this reduction process, **3b** was reacted with FeCp*_2_ which via the 2e reduction process gave intermediate anion **2**, which further led to dimer **1** and free **8**; no paramagnetic species were observed in this process. We continue to study the chemistry of **5b** and still search for its isolable analogues.

## Experimental Section

### General Considerations

All preparations were performed under an anhydrous N_2_ atmosphere by using standard Schlenk and glovebox techniques (Vac.-Atmospheres Nexus II equipped with a −35 °C freezer). Toluene, dichloromethane, and hexane were dried by using a Vac. Atm. Solvent purification system. *o*-Difluorobenzene and CDCl_3_ were dried over CaH_2_ for several days prior to distillation. All solvents were degassed by freeze–pump–thaw and stored on activated 4 Å molecular sieves prior to use. All glassware was oven-dried and cooled under vacuum before use. Commercial reagents were purchased from Sigma-Aldrich, Strem, or Apollo Scientific and used without further purification unless indicated otherwise.

### Spectroscopic Analyses

NMR spectra were recorded at room temperature by using a Bruker AvanceIII-400 MHz spectrometer and referenced to residual solvent, or externally (^11^B: BF_3_·Et_2_O; ^19^F: CFCl_3_; ^31^P: 85% H_3_PO_4_) in some of the cases the tubes were equipped with DMSO-*d*_6_ capillary as external standard. Data for ^1^H NMR are reported as follows: chemical shift (δ ppm), integration, multiplicity (s = singlet, d = doublet, t = triplet, q = quartet, sep = septet, m = multiplet), coupling constant (Hz). The EPR spectra were recorded on a Bruker EMX-10/12 X-band (ν = 9.3 GHz) digital EPR spectrometer equipped with a Bruker N_2_ temperature controller. The spectra were recorded at a microwave power of 100–200 mW and a 100 kHz magnetic field modulation of 0.1–3.0 G amplitude (unless otherwise specified). The digital field resolution was 2048 points per spectrum. This allowed all hyperfine splittings to be measured directly with accuracy better than 0.1 G. Spectra processing and simulation were performed with Bruker WIN-EPR and SimFonia software. When the reactions were performed under UV irradiation, a high-pressure mercury lamp (1000 W) (ARC lamp power supply model 69920) was used, with the output being focused onto the sample with a quartz lens. When the reactions were performed under visible light irradiation (λ = 420–540 nm), a blue LED lamp (34 W) (Kessil, Model No. H150-BLUE) was used.

### Electrochemical Measurements

The cyclic voltammetry (CV) measurements were performed by using a CHI760E electrochemical workstation. A 3 mm glassy carbon was used as the working electrode, Ag wire was used as the reference electrode, and a Pt wire was used as the counter electrode. [nBu_4_N][B(C_6_F_5_)_4_] in CH_2_Cl_2_ (0.1 M) was used as a supporting electrolyte. All electrochemical measurements were performed under an inert atmosphere in a glovebox. All electrodes were rinsed with the electrolyte solution prior to use. For all CVs measurements, the first scan cycle was discarded.

### X-ray Crystallography

Data were collected on a Bruker KAPPA APEX II diffractometer equipped with an APEX II CCD detector using a TRIUMPH monochromator with a Mo Kα X-ray source (α = 0.71073 Å). The crystals were mounted on a cryoloop with Paratone oil, and all data were collected at 100(2) K. Crystal structures were solved by direct methods and refined by full matrix least-squares. All hydrogen atom positions were idealized and rode on the atom of attachment. Structure solution, refinement, graphics, and creation of publication materials were performed by using a SHELXT-2014 and a SHELXL-2014.

### Synthesis of Cp(CO)_3_Mo^•^PPh_2_(*o*-C_2_B_10_H_11_) (**5b**)

Inside the glovebox a J Young NMR tube was charged with [Cp(CO)_3_Mo]_2_ (**1**) (0.05 g, 0.10 mmol) and Ph_2_P(*o*-C_2_B_10_H_11_) (**8**) (0.07 g, 0.2 mmol), and 1 mL of CH_2_Cl_2_ was added. This solution was then placed under irradiation at λ = 420–540 nm in a water bath, and the progress of the reaction was monitored by EPR and NMR spectroscopy. After 1 h, **5b** was measured by EPR spectroscopy, and [Cp(CO)_3_(Ph_2_(*o*-C_2_B_10_H_11_)P)Mo]^+^ (**3b**) and free **8** were measured by ^31^P NMR. CH_2_Cl_2_ was then removed under vacuum, and **5b** was extracted by pentane. Yield: 4%. The EPR spectrum of **5b** was recorded in pentane (Figure 3a). HRMS (APCI): *m*/*z* calcd for C_22_H_25_B_10_P_1_O_3_Mo_1_: 573.1596 (M–H)^−^; found: 573.1174 (Figure 3b).

### Synthesis of [Cp(CO)_3_(Ph_2_(*o*-C_2_B_10_H_11_)P)Mo][B(C_6_F_5_)_4_] (**3b**)

#### Oxidation of **5b** by [Ph_3_C][B(C_6_F_5_)_4_]

Inside the glovebox a J Young NMR tube was charged with **5b** in CH_2_Cl_2_ solution, and a pinch of [Ph_3_C][B(C_6_F_5_)_4_] was added. The EPR spectrum was recorded after 10 min, showing complete disappearance of **5b** and generation of Ph_3_C^•^. ^31^P NMR was recorded after 30 min, showing the formation of **3b** with a typical chemical shift at δ 73.21 ppm.

#### Independent Synthesis

**3b** was synthesized from CpMo(CO)_3_H,^50^ which was prepared by the following procedure: Mo(CO)_6_ (1.00 g, 3.79 mmol) was dissolved in 30 mL of CH_3_CN, and the mixture was refluxed for 12 h. All volatiles were then evaporated under high vacuum, giving the yellow solid Mo(CO)_3_(CH_3_CN)_3_. Mo(CO)_3_(CH_3_CN)_3_ was dissolved in THF (30 mL), and freshly distilled cyclopentadiene (5 mL) was added to this solution and heated for 1 h at 50 °C. After that time, all volatiles were removed, and the remaining solid was sublimed at 60 °C under a high vacuum, giving a yellow crystalline product. [Cp(CO)_3_MoH]_2_ dimer is also formed in this reaction (ca. 10%), as reported in the literature.^50^ The estimated yield for this reaction is ca. 60%. ^1^H NMR (400 MHz; CDCl_3_): δ −5.55 (1H, s, Mo–*H*), 5.42 (5H, s, C_5_*H*_5_). ^13^C NMR (100 MHz; CDCl_3_): δ 90.05 (*C*_5_H_5_), 191.12 and 226.87 (*C*O).

A freshly prepared CpMo(CO)_3_H (0.25 g, 1 mmol) dissolved in 10 mL of CH_2_Cl_2_ was treated with [Ph_3_C][B(C_6_F_5_)_4_] (0.92 g, 1.00 mmol) at −30 °C. The reaction mixture was allowed to warm to room temperature and stirred for another hour, forming a dark violet solution. To this dark violet solution, **8** (0.33 g, 1.00 mmol) dissolved in 5 mL of CH_2_Cl_2_ was added dropwise. The solution was allowed to stir for another hour, turning from violet to red. All the volatiles were evaporated under vacuum, and the residue was washed with (3 × 10) mL of toluene, affording a red solid upon drying. The target compound was crystallized from a CH_2_Cl_2_/benzene (1:10) mixture in 70% yield. ^1^H NMR (400 MHz; *o*-difluorobenzene, DMSO-*d*_6_ capillary): δ 0.99–2.71 (10H, br, B–*H*), 3.31 (1H, s, C–*H*), 4.78 (5H, s, C_5_*H*_5_), 6.88–7.08 (10H, m). ^13^C NMR (100 MHz; CH_2_Cl_2_, DMSO-*d*_6_ capillary): δ 63.19 (cage C–*H*), 69.35 (d, *J*_P,C_ = 18.4 Hz, cage *C*–P), 95.36 (*C*_5_H_5_), 129.53 (d, *J*_P,C_ = 10.9 Hz, Ph), 133.84 (b, Ph), 134.37 (b, *C*_6_F_5_), 136.30 (t, *J*_F,C_ = 13.5 Hz, *C*_6_F_5_), 136.81(b, *C*_6_F_5_), 138.74 (t, *J*_F,C_ = 13.5 Hz, *C*_6_F_5_), 146.25 (b, Ph), 148.64 (b, Ph), 222.76 and 224.04 (*C*O). ^31^P NMR (162 MHz; CH_2_Cl_2_, DMSO-*d*_6_ capillary): δ 73.21 (s). ^19^F NMR (376.5 MHz, CH_2_Cl_2_, DMSO-*d*_6_ capillary): δ −133.96 (b, 8F), −164.49 (t, 4F, *J* = 20.1 Hz), −168.36 (b, 8F). ^11^B NMR (128 MHz; CH_2_Cl_2_, DMSO-*d*_6_ capillary): δ −0.17, −1.50, −2.62, −7.91, −12.68, −17.37. HRMS (ESI^+^): *m*/*z* calcd for C_22_H_26_B_10_P_1_O_3_Mo_1_: 574.1713 (M^+^); found: 574.1708.

### Synthesis of Ph_2_P(*o*-C_2_B_10_H_11_) (**8**)^31,32^

*o*-Carborane (1.00 g, 6.93 mmol) dissolved in 50 mL of dimethoxyethane (DME) was reacted with *n*-BuLi in hexane (2.91 mL, 7.28 mmol) at −15 °C and stirred at this temperature for 1 h. After that time the reaction mixture was allowed to warm to room temperature and stirred for another hour. A 10 mL dimethoxyethane solution of chlorodiphenylphosphine (1.28 mL, 6.93 mmol) was added to the stirring solution at −15 °C. The solution was allowed to warm to room temperature and stirred for 1 h followed by 1 h reflux. All the volatiles were evaporated, and the residue was extracted with Et_2_O which afforded a white solid upon drying. The target compound was purified by column chromatography on silica gel (60–200 mesh) eluted with CH_2_Cl_2_–hexane (1:5). Yield: 80%. ^1^H NMR (400 MHz; CDCl_3_), δ 1.75–2.86 (10H, br, B–*H*), 3.53 (1H, s, C–*H*), 7.49–7.54 (6H, m), 7.81 (4H, m). ^13^C NMR (100 MHz; CDCl_3_): δ 63.6 (d, *J*_P,C_ = 15.4 Hz, cage C–*H*), 72.78 (d, *J*_P,C_ = 75.85 Hz, cage *C*–P), 128.85 (d, *J*_P,C_ = 9.6 Hz, Ph), 131.23 (s, Ph), 131.98 (d, *J*_P,C_ = 15.92 Hz, Ph), 134.99 (d, *J*_P,C_ = 26.54 Hz, Ph). ^31^P NMR (162 MHz; CDCl_3_): δ 25.02 (s). ^11^B NMR (128 MHz; CDCl_3_), δ −1.26, −2.38, −6.92, −8.10, −9.83, −11.69, −12.96, −14.15, −15.39.

### Synthesis of Cp(Cl)(CO)_2_MoPPh_2_(*o*-C_2_B_10_H_11_) (**9**)

The pentane solution of **5b** was reacted with an excess of Ph_3_CCl. The EPR spectrum was recorded right after, showing almost complete disappearance of **5b** and formation of Ph_3_C^•^ (see Figure S22). Overnight red crystals of **9** were formed from this solution in 92% yield. Noteworthy, **9** is not stable in CHCl_3_ or C_6_H_6_ solutions for a long period of time. ^1^H NMR (400 MHz; CDCl_3_): δ 1.65–3.35 (10H, br, B–*H*), 4.60 (1H, s, C–*H*), 5.23 (5H, s, C_5_*H*_5_), 7.45–7.49 (5H, m), 7.57–7.58 (1H, m), 7.69–7.73 (2H, m), 8.06 (2H, t, *J* = 9.12 Hz). ^13^C NMR (100 MHz; CDCl_3_): δ 66.46 (d, *J*_P,C_ = 8.6 Hz, cage C–*H*), 95.56 (*C*_5_H_5_), 127.69 (d, *J*_P,C_ = 9.6 Hz, Ph), 127.75 (d, *J*_P,C_ = 9.6 Hz, Ph), 130.92, 132.47, 133.06 (d, *J*_P,C_ = 10.2 Hz, Ph), 136.75 (d, *J*_P,C_ = 11.5 Hz, Ph). ^31^P NMR (162 MHz; CDCl_3_): δ 70.69 (s). ^11^B NMR (128 MHz; CDCl_3_): δ 0.74, −0.21, −1.42, −2.91, −4.2, −7.29, −8.42, −11.86, −12.93. HRMS (ESI^+^): *m*/*z* calcd for C_21_H_26_B_10_P_1_O_2_Mo_1_: 547.1727 (M–Cl)^+^; found: 547.1728.

## Supporting Information Available

NMR, EPR, CV, experimental and computational details (PDF)

## Accession Codes

CCDC 2057570–2057572 contain the supplementary crystallographic data for this paper. These data can be obtained free of charge via www.ccdc.cam.ac.uk/data_request/cif, or by emailing data_request@ccdc.cam.ac.uk, or by contacting The Cambridge Crystallographic Data Centre, 12 Union Road, Cambridge CB2 1EZ, UK; fax: +44 1223 336033.
