# Supplementary material for: Carborane Stabilized “19-Electron” Molybdenum Metalloradical
Source: J Am Chem Soc. 2021 Jun 23;143(26):9842–8. doi: 10.1021/jacs.1c03568 (PMC8397321; doi:10.1021/jacs.1c03568)
Supplement: Supplementary file 1 — ja1c03568_si_001.pdf [file ja1c03568_si_001.pdf]

Supporting Information for

Carborane Stabilized "19-Electron" Molybdenum Metalloradical

Kuldeep Jaiswal,[a] Naveen Malik,[b] Boris Tumanskii,*[a] Gabriel Ménard[c] and Roman Dobrovetsky*[a]

† School of Chemistry, Raymond and Beverly Sackler Faculty of Exact Sciences, Tel Aviv University, Tel Aviv 69978, Israel

†† Department of Organic Chemistry, Weizmann Institute of Science Rehovot 7610001, Israel

‡ Department of Chemistry and Biochemistry, University of California, Santa Barbara, California 93106, United States

Email: rdobrove@tau.ac.il, tboris@tau.ac.il

Experimental Details………………………………………………………………………………. S2

NMR Spectra ……………………………………………………………………………….….S7

X-Ray molecular structure of $[CoCp^*_2][B(C_6F_5)_4]$………………………………………………...S18

EPR Spectra of the reaction of **5b** with Ph_3CCl ..……………………………………………...S18

EPR and Mass Spectra of C_{60} trap experiments…………………………………………………S19

CV experiments ……………………………………………………………………………...S21

UV-VIS spectra of Compound **3b**..........…………………………………………………………S25

IR spectra of Compound **5b** and **3b**………………………………………………………….S26

Computational Studies ………………………………………………………………………...S27

References ……………………………………………………………………………………...S42

Experimental

Scheme S1. General scheme of the reactions done in this work.

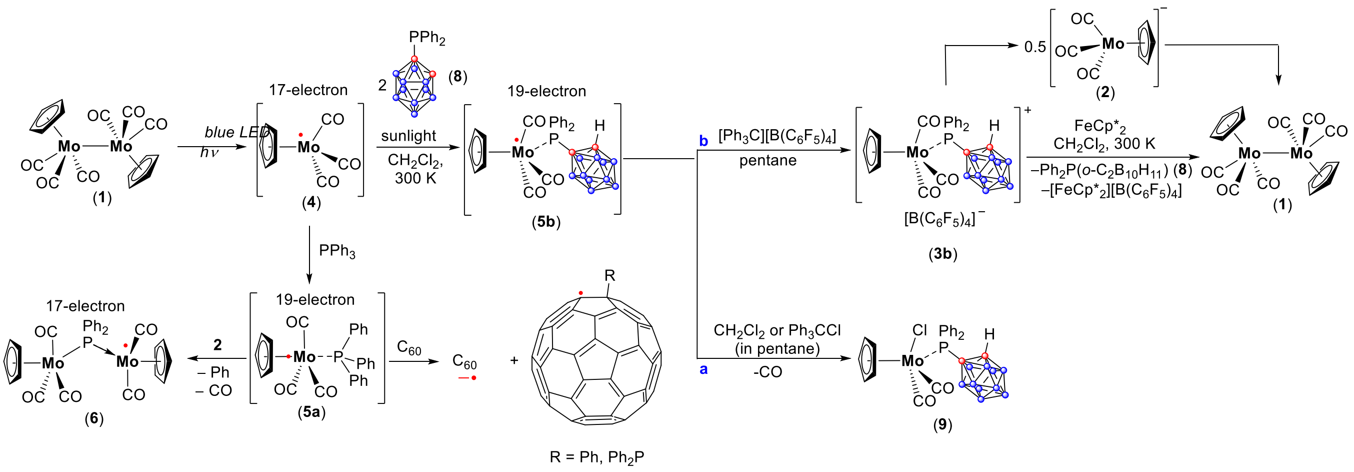

General information

Commercial reagents were purchased from Sigma Aldrich, Strem or Apollo Scientific and used without further purification unless indicated otherwise. NMR spectra were recorded at room temperature using a Bruker AvanceIII-400 MHz spectrometer. Data for ^1H NMR are reported as follows: chemical shift (δ ppm), integration, multiplicity (s = singlet, d = doublet, t = triplet, q = quartet, quin = quintet, m = multiplet, br = broad), coupling constant (Hz), assignment. UV-VIS spectra was recorded on a TECAN Infinite M200Pro spectrometer using quartz cuvettes. FTIR spectra were recorded using an ATR unit on a Bruker Tensor 27 spectrometer (Germany).

Prior to the EPR experiments, the samples were degassed by 3-fold freeze-thaw procedures. The EPR spectra were recorded on a Bruker EMX-10/12 X-band (v= 9.3 GHz) digital EPR spectrometer equipped with a Bruker N_2-temperature controller. The spectra were recorded at a microwave power of 100-200 mW, 100 kHz magnetic field modulation of 1.0-3.0 G amplitude. Digital field resolution was 2048 points per spectrum. This allowed all hyperfine splitting to be measured directly with accuracy better than 0.2 G. Spectra processing and simulation were performed with the Bruker WIN-EPR and SimFonia Software. The g-factor values were determined using 2,2,6,6-tetramethylpiperidine-N-oxyl (TEMPO) as a reference (g=2.0058). When the reactions were carried out under UV irradiation, a high-pressure mercury lamp (1 kW) (ARC lamppower supply model 69920) was used, with the output being focused onto the sample

with a quartz lens and filtered through distilled water to remove infrared radiation. When the reactions were carried out under visible light irradiation (λ = 420-540 nm), a blue LED lamp (34 W) (Kessil, Model No. H150-BLUE) was used.

Atmospheric pressure chemical ionization (APCI) mass spectra were obtained with the use instrument represented a Bruker maXis impact QTOFmass spectrometer operated in the APCI mode. Nitrogen was used as both nebulizer (0.16 MPa) and drying (4.0 L min^{-1}) gases. The temperature of the APCI heater was 350 °C and 200 °C of the drying heater. The corona discharge current was 2 μA.

Single Crystal X-ray Analysis Deposition Numbers 2057570- 2057572 contain the supplementary crystallographic information for this work. These data are provided free of charge by the joint Cambridge Crystallographic Data Centre and Fachinformationszentrum Karlsruhe Access Structures service under www.ccdc.cam.ac.uk/structures.

Electrochemical measurements.

The cyclic voltammograms (CVs) measurements were carried out using a CHI760E electrochemical workstation. Where, 3 mm glassy carbon, as the working electrode, Ag wire was used as the reference electrode, and a Pt wire as the counter electrode, respectively. $[nBu_4N][B(C_6F_5)_4]$ (TBABARF) in CH_2Cl_2 (0.1 M) was used as a supporting electrolyte. All electrochemical measurements were performed under an inert atmosphere in glove box. All electrodes and were rinsed with the electrolyte solution prior to use. For all CVs measurements, the first scan cycle was discarded.

Synthesis of 8[1a]**:** Ortho-carborane (1.00 g, 6.93 mmol) dissolved in 50 mL of dimethoxyethane (DME),

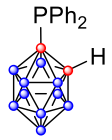

was reacted with n-BuLi in hexane (2.91 mL, 7.28 mmol) at -15 °C and stirred at this temperature for 1 h. After that time the reaction mixture was allowed to warm to room temperature and stirred for another hour. A 10 mL DME solution of chlorodiphenylphosphine (1.28 mL, 6.93 mmol) was added to the stirring solution at -15 °C. The solution was allowed to warm to room temperature and stirred for 1 h followed by 1 h reflux. All the volatiles were evaporated and the residue was extracted with Et_2O that afforded a white solid upon drying. The target compound was purified by column chromatography on silica gel (60-200 mess) eluted with CH_2Cl_2−hexane (1:5). Yield: 80%. ^{1}H NMR (400 MHz; $CDCl_3$), δ 1.75-2.86 (10H, br, B-*H*), 3.53 (1H, s, C-*H*), 7.49-7.54 (6H, m), 7.81 (4H, m). ^{13}C NMR (100 MHz; $CDCl_3$), δ 63.6 (d, $J_{P,C,}$= 15.4 Hz, cage C-*H*), 72.78 (d, $J_{P,C}$ = 75.85 Hz, cage *C*-P), 128.85 (d, $J_{P,C}$ = 9.6 Hz, Ph), 131.23 (s, Ph), 131.98 (d, $J_{P,C}$ = 15.92 Hz, Ph), 134.99 (d, $J_{P,C}$ =

26.54 Hz, Ph). ^{31}P NMR (162 MHz; $CDCl_3$) δ 25.02 (s). ^{11}B NMR (128 MHz; $CDCl_3$), δ -1.26, -2.38, -6.92, -8.10, -9.83, -11.69, -12.96, -14.15, -15.39.

Synthesis of 5b: Inside the glovebox a J Young NMR tube was charged with $[Cp(CO)_3Mo]_2$ (**1**) (0.05 g, 0.10 mmol) and $Ph_2P(o\text{-}C_2B_{10}H_{11})$ (**8**) (0.07 g, 0.2 mmol) and 1 mL CH_2Cl_2 was added. This solution was then placed under irradiation at λ = 420-530 nm in a water bath (or under sunlight for 2 days) and the progress of the reaction was monitored by EPR and NMR spectroscopy. After 2 days **5b** was measured by EPR spectra, and $[Cp(CO)_3(Ph_2(o\text{-}C_2B_{10}H_{11})P)Mo]^+$ and free **8** were measured by ^{31}P NMR. Importantly, the NMR spectra recorded after storing this sample in dark for 3 days showed that mixture converted back to starting material (**8**). Repeating the sunlight irradiation for another 3 days gave the regeneration of **5b** and $[Cp(CO)_3(Ph_2(o\text{-}C_2B_{10}H_{11})P)Mo]^+$ (see Figure S20). Yield: 4%.

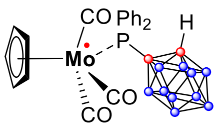

Synthesis of 9: 5b was generated by the method described above, from $[Cp(CO)_3Mo]_2$ (**1**) (0.13 g, 0.26 mmol) and $Ph_2P(o\text{-}C_2B_{10}H_{11})$ (**8**) (0.17 g, 0.51mmol) and 30 mL CH_2Cl_2, followed by evaporation of the volatiles and extraction with pentane. The pentane solution of **5b** was reacted with an excess of Ph_3CCl. The EPR spectra was recorded right after, showing almost complete disappearance of **5b** and formation of $Ph_3C^{\bullet}$ (see Figure S24). Overnight red crystals of **9** were formed from this solution. Noteworthy, **9** is not stable in $CHCl_3$ or C_6H_6 solutions for a long period of time. ^{1}H NMR (400 MHz; $CDCl_3$), δ 1.65-3.35 (10H, br, B-*H*), 4.60 (1H, s, C-*H*), 5.23 (5H, s, C_5H_5), 7.45-7.49 (5H, m), 7.57-7.58 (1H, m), 7.69-7.73 (2H, m), 8.06 (2H, t, J = 9.12 Hz). ^{13}C NMR (100 MHz; $CDCl_3$), δ 66.46 (d, $J_{P,C}$ = 8.6 Hz, cage C-*H*), 95.56 (C_5H_5), 127.69 (d, $J_{P,C}$ = 9.6 Hz, Ph), 127.75 (d, $J_{P,C}$ = 9.6 Hz, Ph), 130.92, 132.47, 133.06 (d, $J_{P,C}$ = 10.2 Hz, Ph), 136.75 (d, $J_{P,C}$ = 11.5 Hz, Ph). ^{31}P NMR (162 MHz; $CDCl_3$) δ 70.69 (s). ^{11}B NMR (128 MHz; $CDCl_3$), δ 0.74, -0.21, -1.42, -2.91, -4.2, -7.29, -8.42, -11.86, -12.93. HRMS (ESI$^+$): m/z calc'd for $C_{21}H_{26}B_{10}P_1O_2Mo_1$: 547.1727 $(M-Cl)^+$; found: 547.1728.

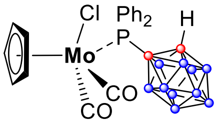

Oxidation of 5b by $[Ph_3C][B(C_6F_5)_4]$ - formation of 3b : Inside the glovebox a J Young NMR tube was charged with **5b** in CH_2Cl_2 solution and a pinch of $[Ph_3C][B(C_6F_5)_4]$ was added. The EPR spectra was recorded after 10 min., showing complete disapperance of **5b** and generation of $Ph_3C^{\bullet}$. ^{31}P NMR was recorded after 30 min. showing the

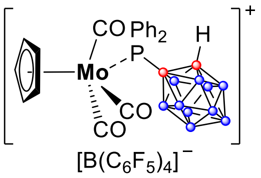

formation of **3b** with a typical chemical shift at δ 73.21 ppm, which was confirmed by the indepedent synthesis of **3b** (described below).

Synthesis of 3b: 3b was synthesized from $CpMo(CO)_3H$,[1b] which was prepared by following procedure: $Mo(CO)_6$ (1.00 g, 3.79 mmol) was dissolved in 30 mL of CH_3CN, and the mixture was refluxed for 12 h. All volatiles were then evaporated under high vacuum, giving yellow solid $Mo(CO)_3(CH_3CN)_3$. $Mo(CO)_3(CH_3CN)_3$ was dissolved in THF (30 mL) and freshly distilled cyclopentadiene (5 mL) was added to this solution and heated for 1 h at 50 °C. After that time, all volatiles were removed, and the remaining solid was sublimed at 60 °C under high vacuum giving yellow crystaline product. $[Cp(CO)_3MoH]_2$ dimer is also formed in this reaction (ca. 10%), as reported in literature.[1b] The estimated yield for this reaction is ca. 60%. ^1H NMR (400 MHz; $CDCl_3$), δ -5.55 (1H, s, Mo-*H*), 5.42 (5H, s, C_5H_5). ^{13}C NMR (100 MHz; $CDCl_3$), δ 90.05 (C_5H_5), 191.12 and 226.87 (*C*O).

A freshly prepared $CpMo(CO)_3H$ (0.25 g, 1 mmol) dissolved in 10 mL of CH_2Cl_2 was treated with $[Ph_3C][B(C_6F_5)_4]$ (0.92 g, 1.00 mmol) at −30 °C. The reaction mixture was allowed to warm to room temperature and stirred for another hour, forming a dark violet solution. To this dark violet solution, **8** (0.33 g, 1.00 mmol) dissolved in 5 mL of CH_2Cl_2 was added dropwise. The solution was allowed to stir for another hour, turning from violet to red. All the volatiles were evaporated under vacuum and the residue was washed with (3 x 10) mL toluene, affording a red solid upon drying. The target compound was crystalized from CH_2Cl_2/Benzene (1:10) mixture in 70% yield. ^1H NMR (400 MHz; o-difluorobenzene, DMSO-d_6 capillary), δ 0.99-2.71 (10H, br, B-*H*), 3.31 (1H, s, C-*H*), 4.78 (5H, s, C_5H_5), 6.88-7.08 (10H, m). ^{13}C NMR (100 MHz; CH_2Cl_2, DMSO-d_6 capillary), δ 63.19 (cage C-*H*), 69.35 (d, $J_{P,C}$ = 18.4 Hz, cage *C*-P), 95.36 (C_5H_5), 129.53 (d, $J_{P,C}$ = 10.9 Hz, Ph), 133.84 (b, Ph), 134.37 (b, C_6F_5), 136.30 (t, $J_{F,C}$ = 13.5 Hz, C_6F_5), 136.81(b, C_6F_5), 138.74 (t, $J_{F,C}$ = 13.5 Hz, C_6F_5), 146.25 (b, Ph), 148.64 (b, Ph), 222.76 and 224.04 (*C*O). ^{31}P NMR (162 MHz; CH_2Cl_2, DMSO-d_6 capillary), δ 73.21 (s). ^{19}F NMR (376.5 MHz, CH_2Cl_2, DMSO-d_6 capillary), δ -133.96 (b, 8F), -164.49 (t, 4F, J = 20.1 Hz), -168.36 (b, 8F). ^{11}B NMR (128 MHz; CH_2Cl_2, DMSO-d_6 capillary), δ -0.17, -1.50, -2.62, -7.91, -12.68, -17.37. HRMS (ESI^+): m/z calc'd for $C_{22}H_{26}B_{10}P_1O_3Mo_1$: 574.1713 (M^+); found: 574.1708.

Generation of 5a: Inside the glovebox a J Young NMR tube was charged with $[Cp(CO)_3Mo]_2$ (**1**) (0.05 g, 0.1 mmol) and Ph_3P (0.06 g, 0.2 mmol) and 1 mL CH_2Cl_2 was added. The EPR and NMR spectra were recorded after exposing this solution to sunlight for 5h and 5 days, respectively (see figure **21** for NMR).

Reaction of 3b with Cp^*_2M (M = Fe, Co): Inside the glovebox a J Young NMR tube was charged with (**3b**) (0.13 g, 0.10 mmol) and Cp^*_2M (0.03 g, 0.10 mmol) and 1 mL CH_2Cl_2 was added. The EPR and NMR spectra were recorded immediately after. The EPR spectra we did not show the formation of any paramagnetic species. ^{31}P NMR spectra showed **3b** and **8** in 1:1 ratio (Figure S22)**.** Overtime, **3b** was completely consumed and only **8** could be seen in ^{31}P NMR. The reaction mixture of **3b** and Cp^*_2Co, volatiles were evaporated and **8** was washed out by hexane leaving a red solid. The solid was washed with CH_3CN, slow evaporation of CH_3CN solution gave a new polymorph crystals of a byproduct $[Cp^*_2Co^{III}][B(C_6F_5)_4]$,[4] which molecular structure was determined by X-ray crystallography (Figure S24). The remaining solid after CH_3CN wash was redissolved in CH_2Cl_2 and crystallized giving compound **1**.

Reactions in presence of C_{60}

To support the suggested photoinduced Ph−P bond cleavage in transient 19-e **5a** (Scheme S1), the reaction between **1** and Ph_3P was performed in C_{60} saturated toluene solution, which is a very efficient radical trap.[2] As a result, under UV-irradiation we almost immediately observed a superposition of two signals in EPR spectra. The first signal corresponding to the typical signal of $C_{60}^{\bullet-}$,[3] the second smaller signal corresponding to radical adducts to C_{60} (Figure S25, a). Investigation of the reaction mixture using MS showed masses related to the adducts of $Ph^\bullet$ and $Ph_2P^\bullet$ radicals to C_{60} (Figure S25, b and c). The formation of the C_{60} radical anion indicates the reduction of C_{60} by the 19-e complex **5a**, which again shows the strong reducing nature of 19-e Mo-centered radical species and more specifically of **5a**. Furthermore, the fixation of the addition of $Ph^\bullet$ to C_{60} indicates the decomposition of the 19-e complex **5a** under light via cleavage of the Ph−P bond. Importantly, in a similar control experiment of the UV-irradiation of a toluene solution of **5b** and C_{60} (1:10), neither $C_{60}^{\bullet-}$ nor adducts of $Ph^\bullet$ addition to C_{60} were found, which means that **5b** is significantly less reducing compared to **5a**, as well as, more stable under irradiation.

Figure S1. ^{1}H NMR of **8**

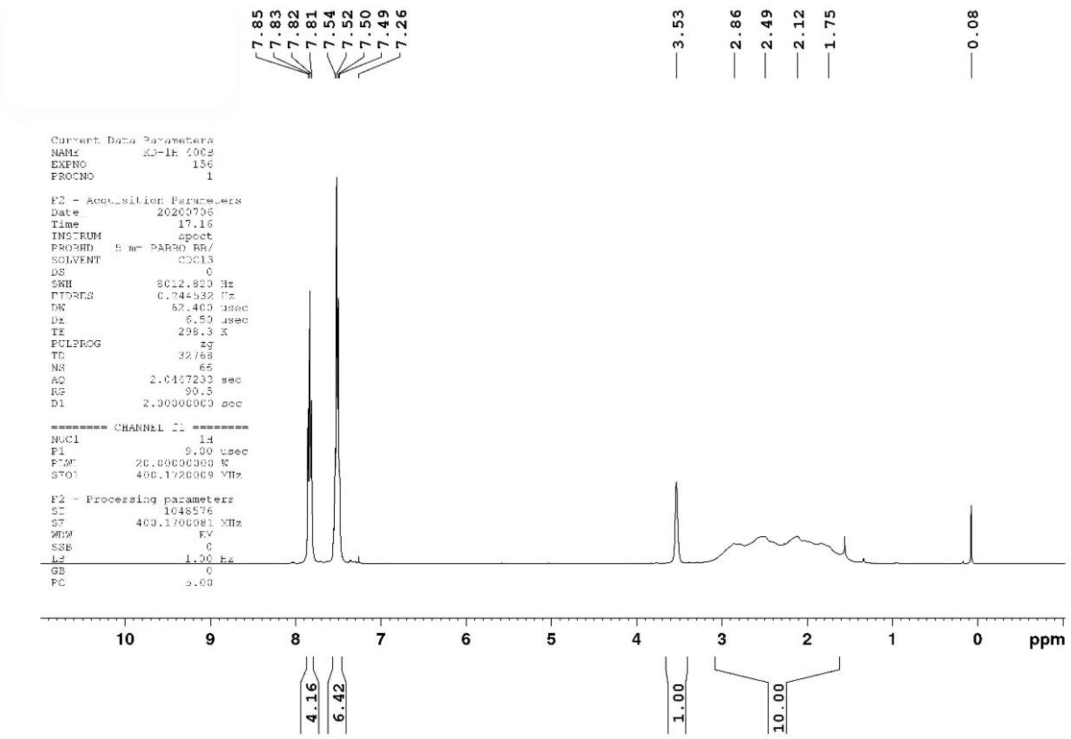

Figure S2. ^{13}C NMR of **8**

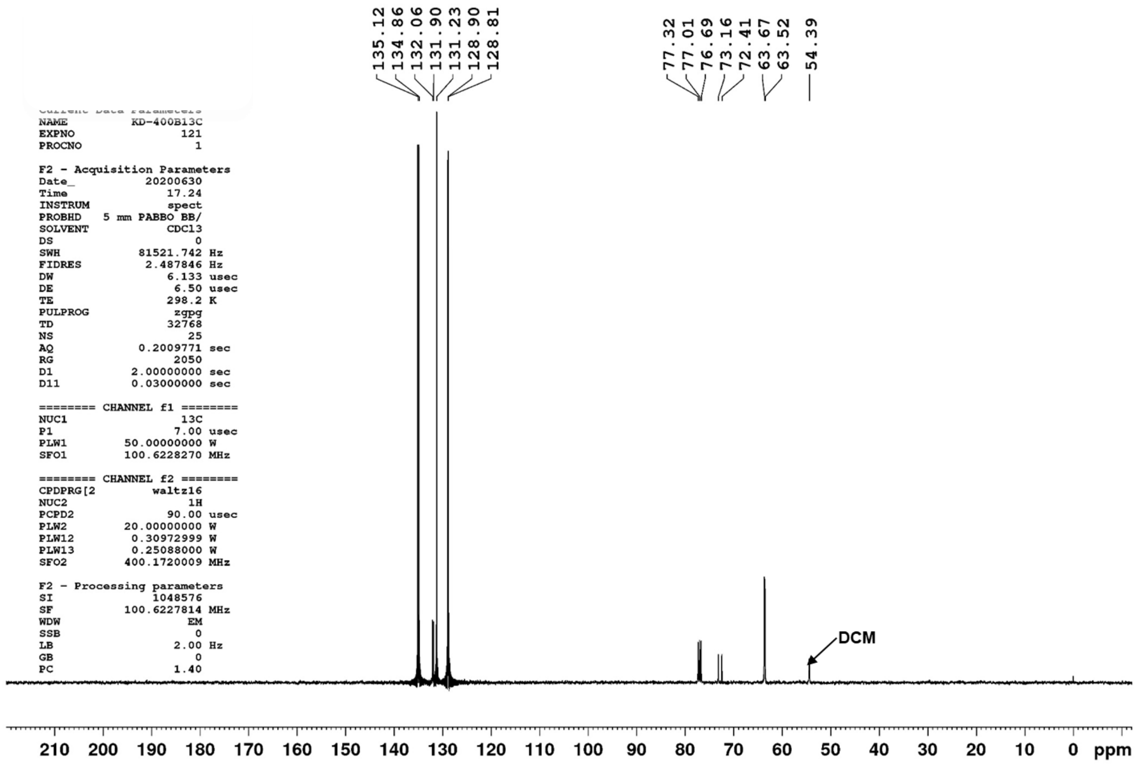

Figure S3. ^{31}P NMR of **8**

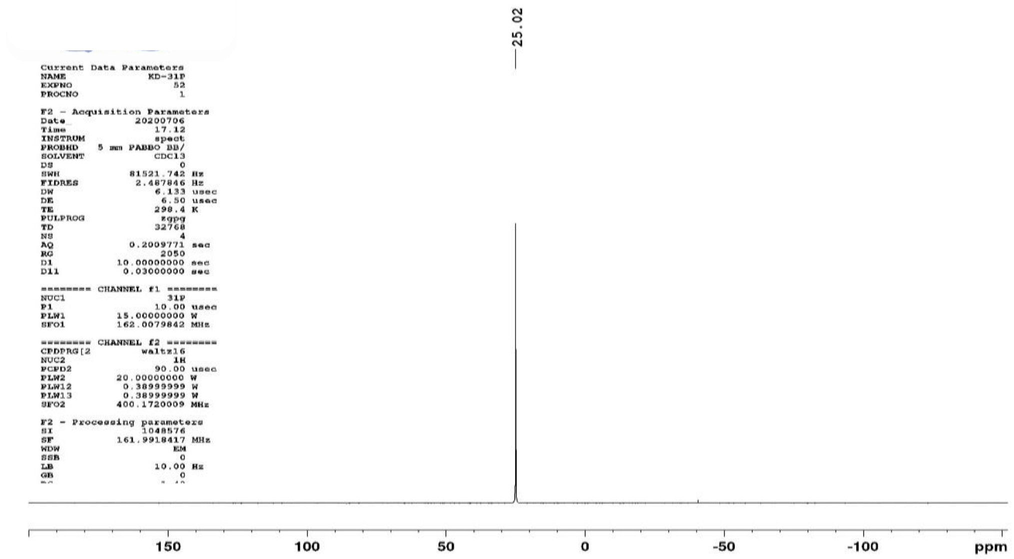

Figure S4. ^{11}B NMR of **8**

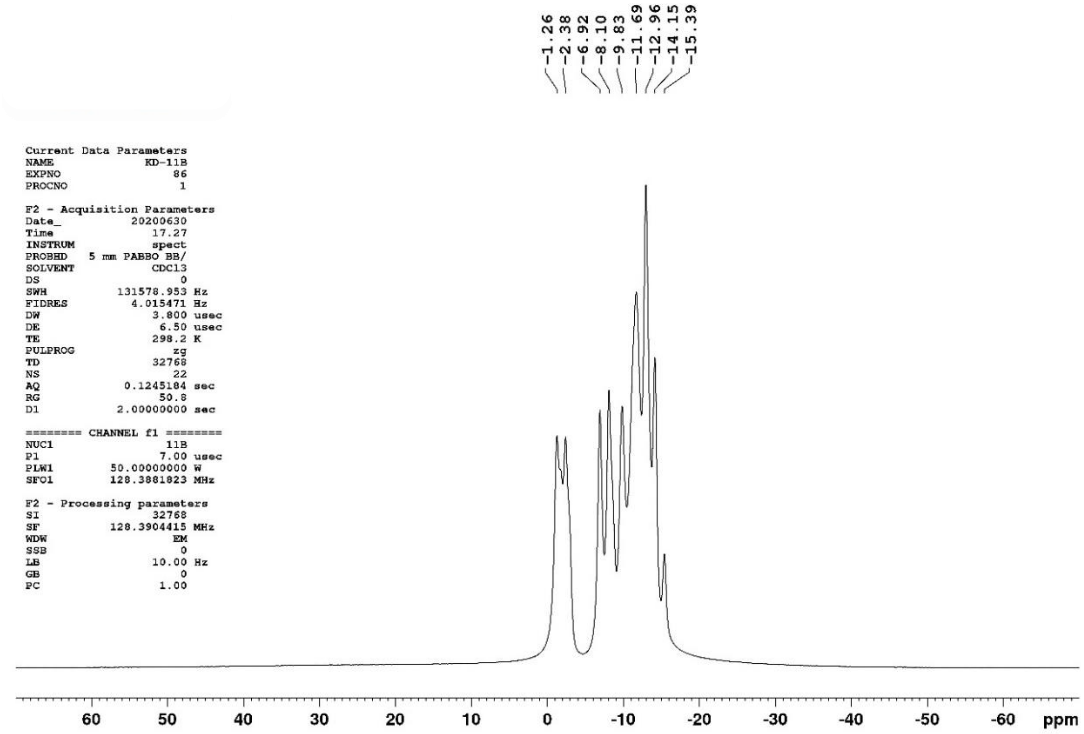

Figure S5. ^{1}H NMR of $CpMo(CO)_3H$

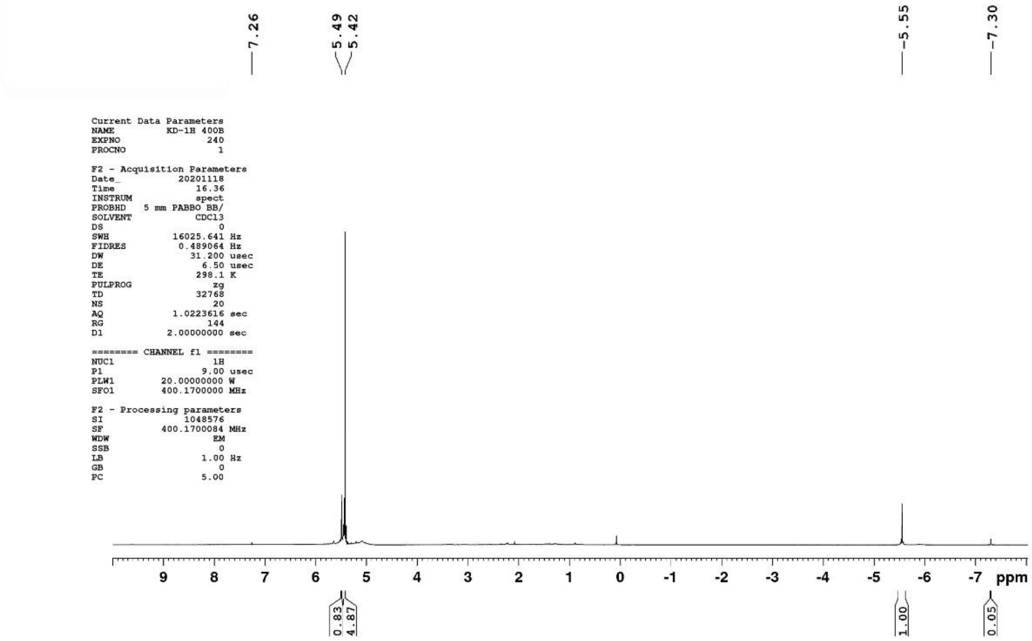

Figure S6. ^{13}C NMR of $CpMo(CO)_3H$

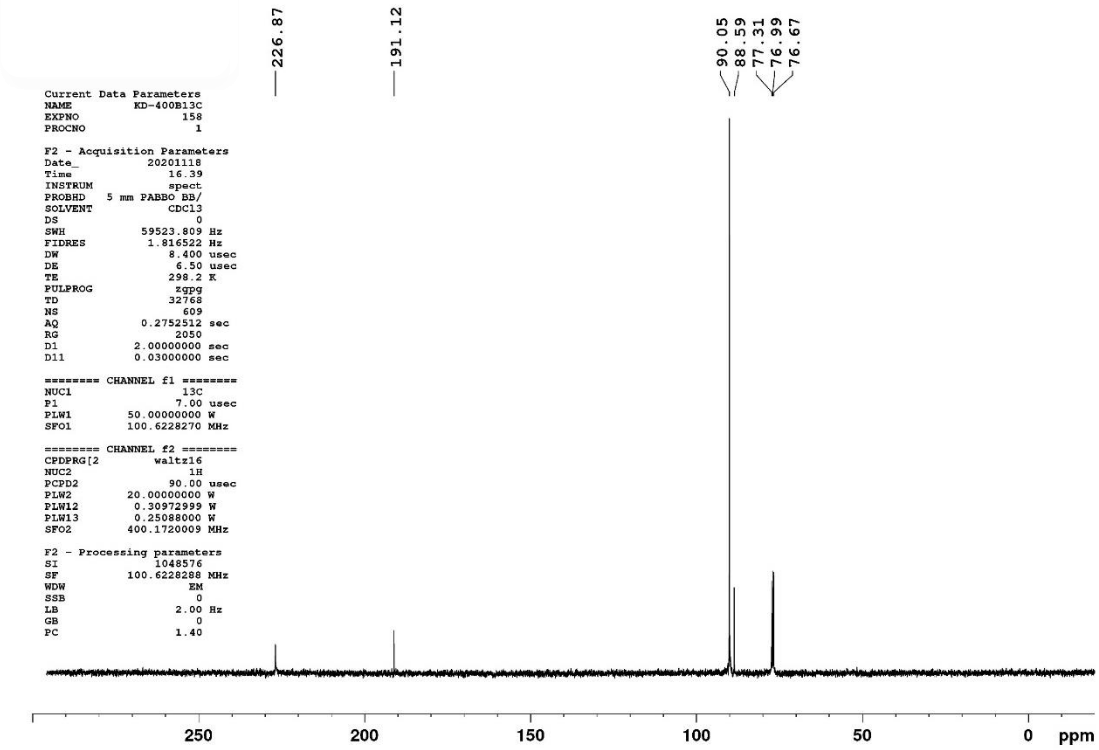

Figure S7. ^{1}H NMR of **9**

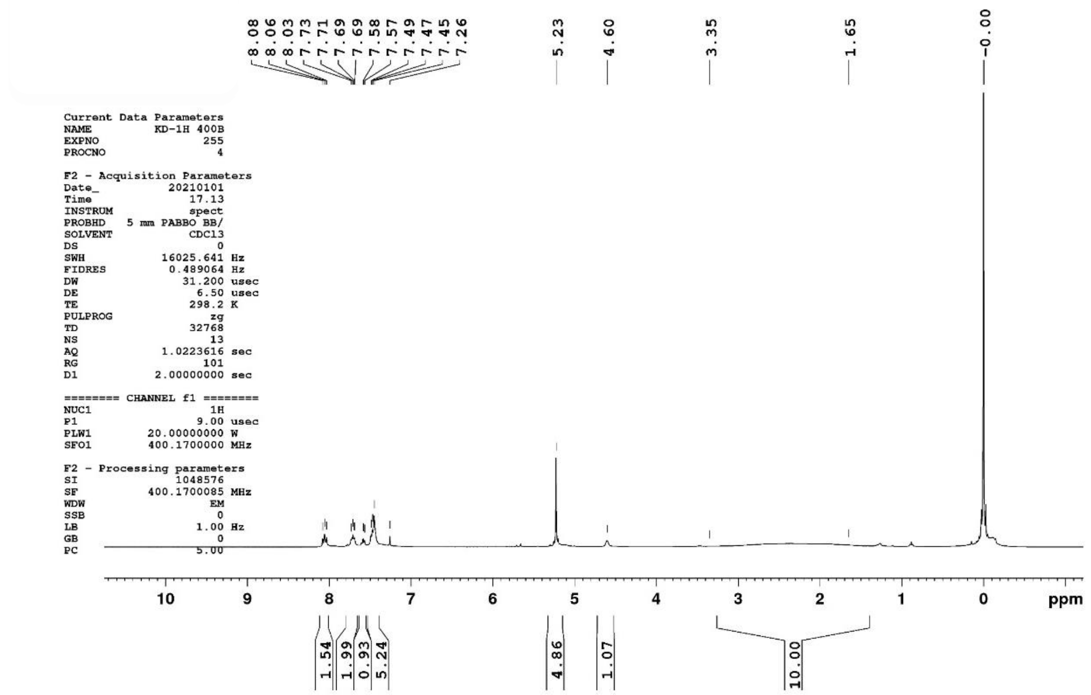

Figure S8. ^{1}H{^{11}B} NMR of **9**

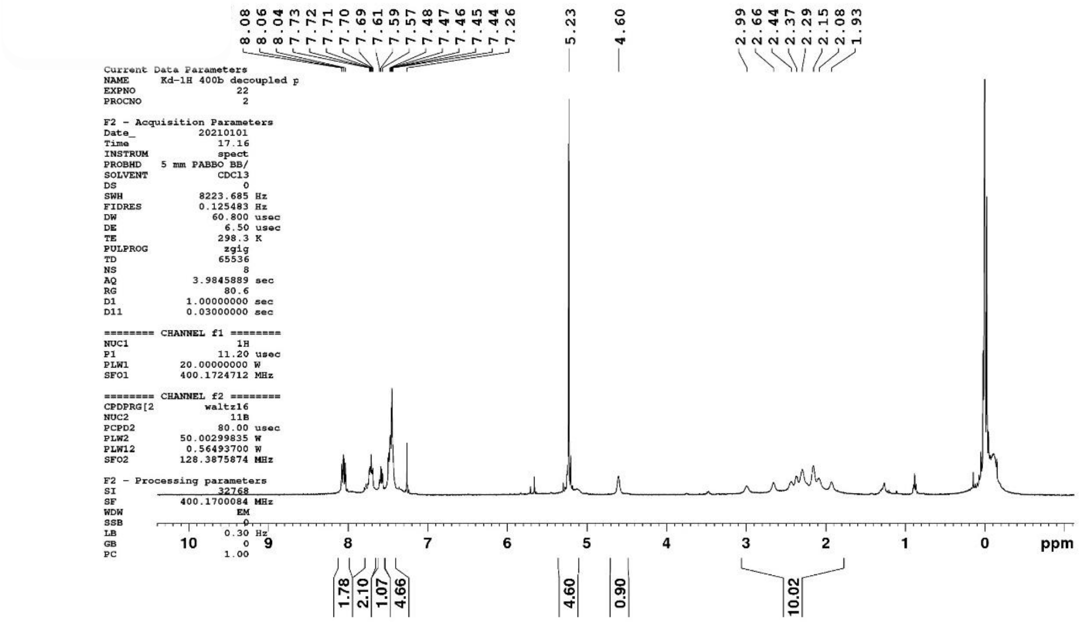

Figure S9. ^{31}P NMR of **9**

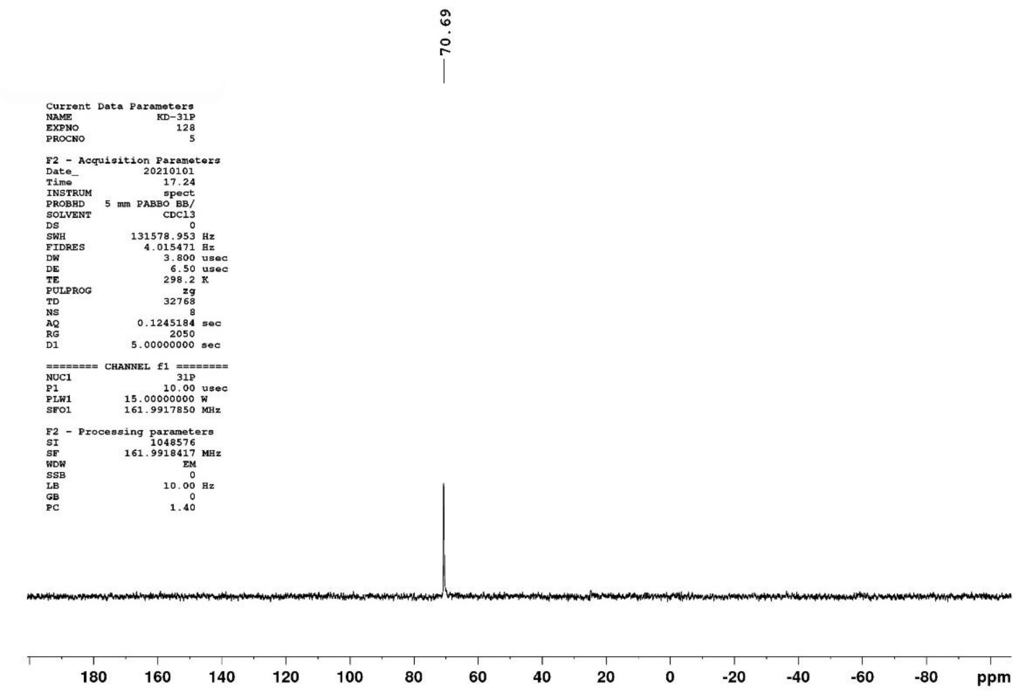

Figure S10. ^{13}C NMR of **9**

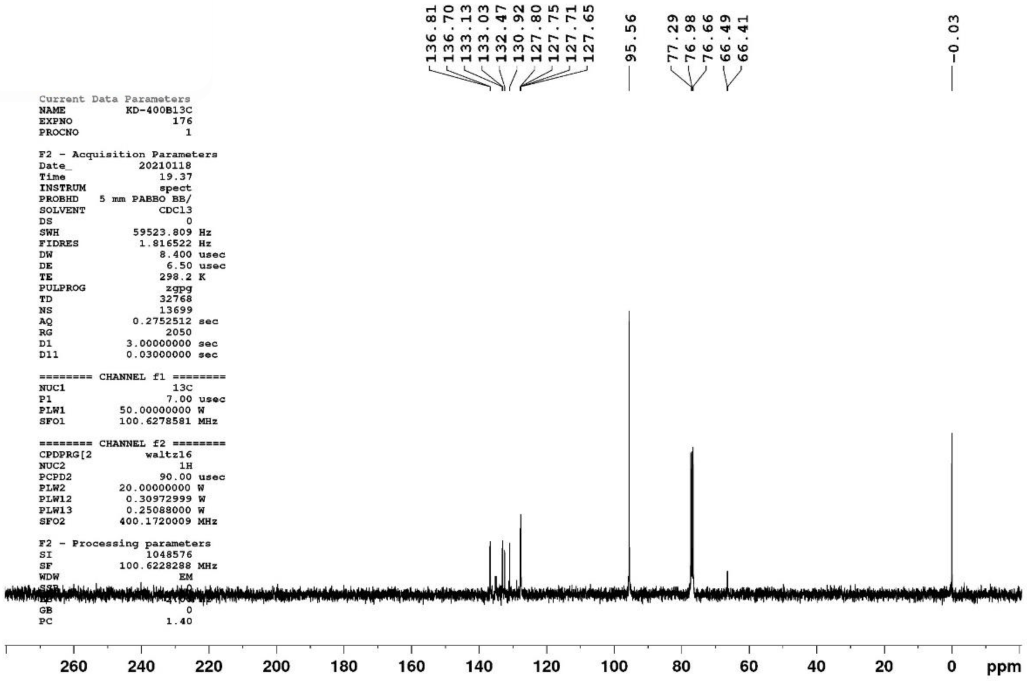

Figure S11. ^{11}B NMR of **9**

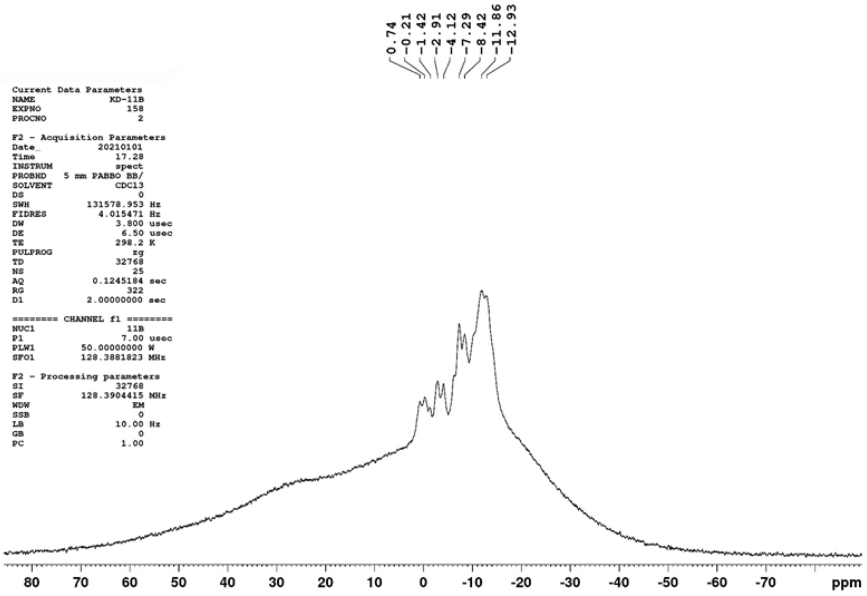

Figure S12. ^{11}B{^{1}H} NMR of **9**

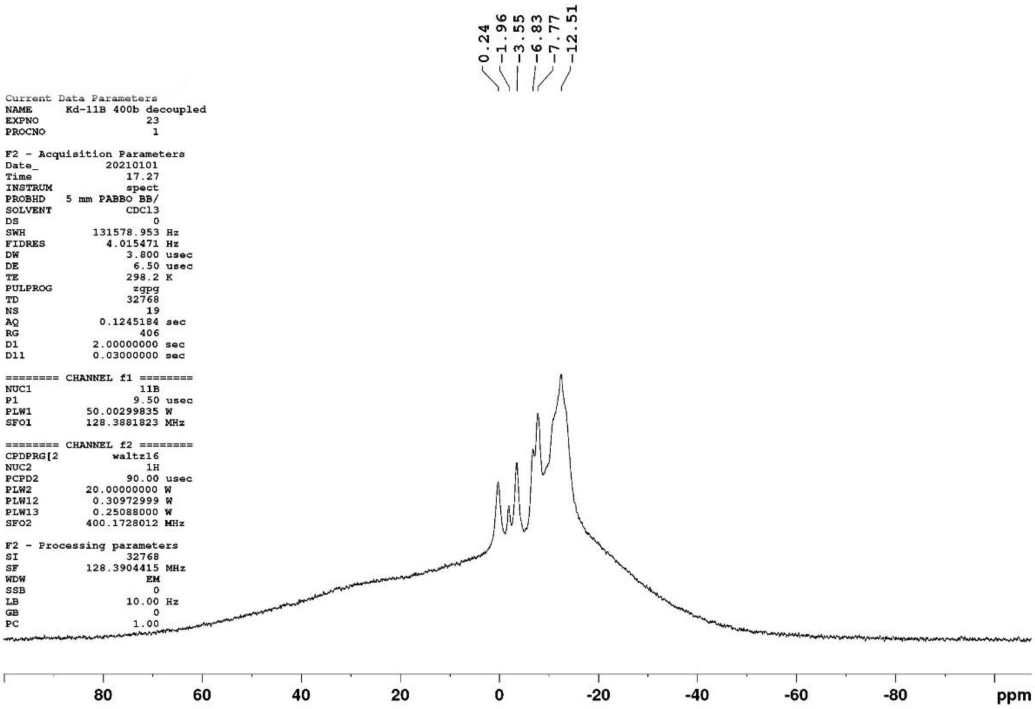

Figure S13. ^{1}H NMR of **3b**

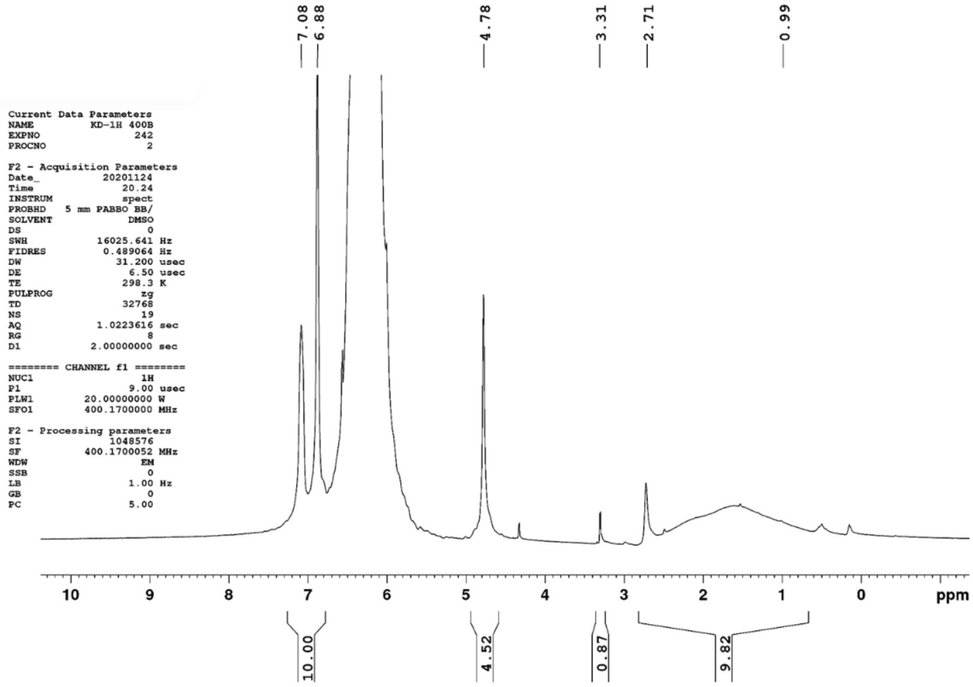

Figure S14. ^{1}H{^{11}B} NMR of **3b**

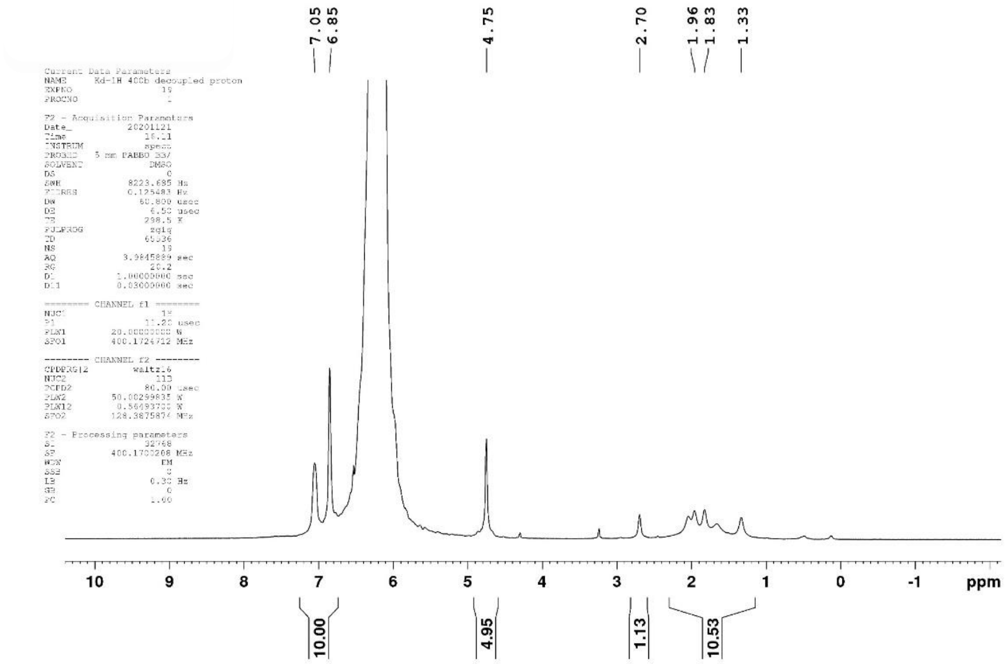

Figure S15. ^{13}P NMR of **3b**

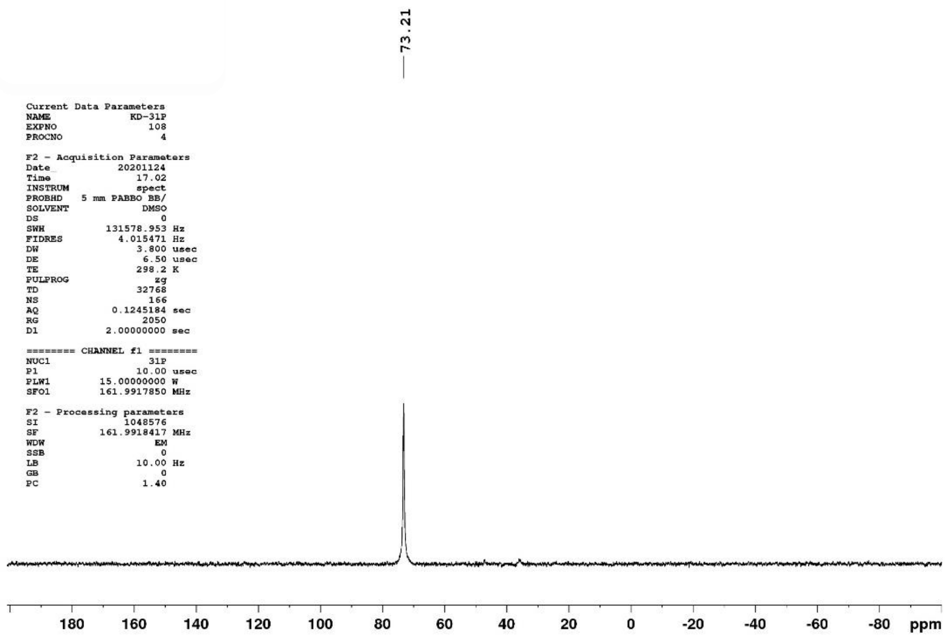

Figure S16. ^{13}C NMR of **3b**

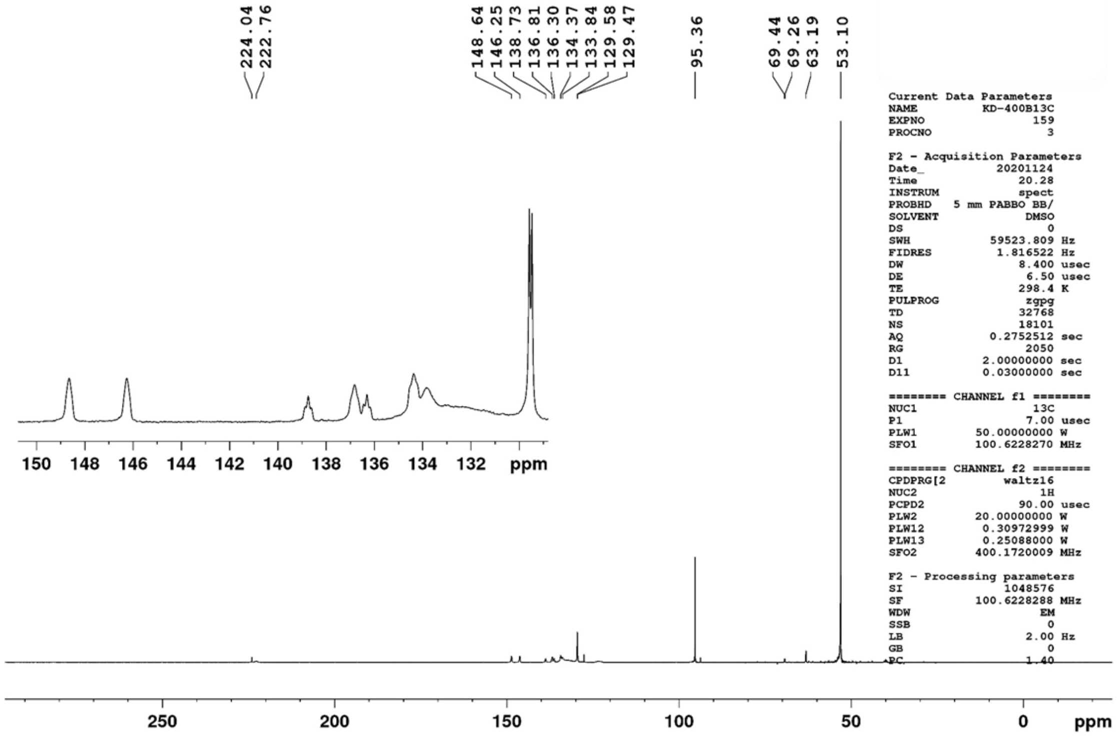

Figure S17. ^{11}B NMR of **3b**

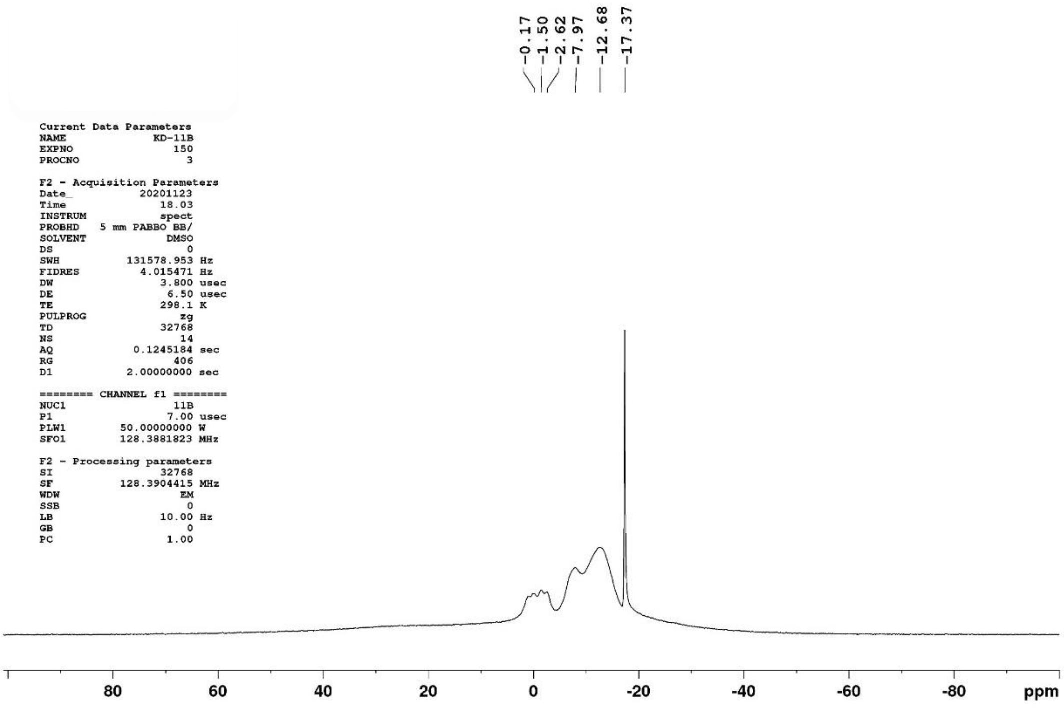

Figure S18. ^{11}B{^{1}H} NMR of **3b**

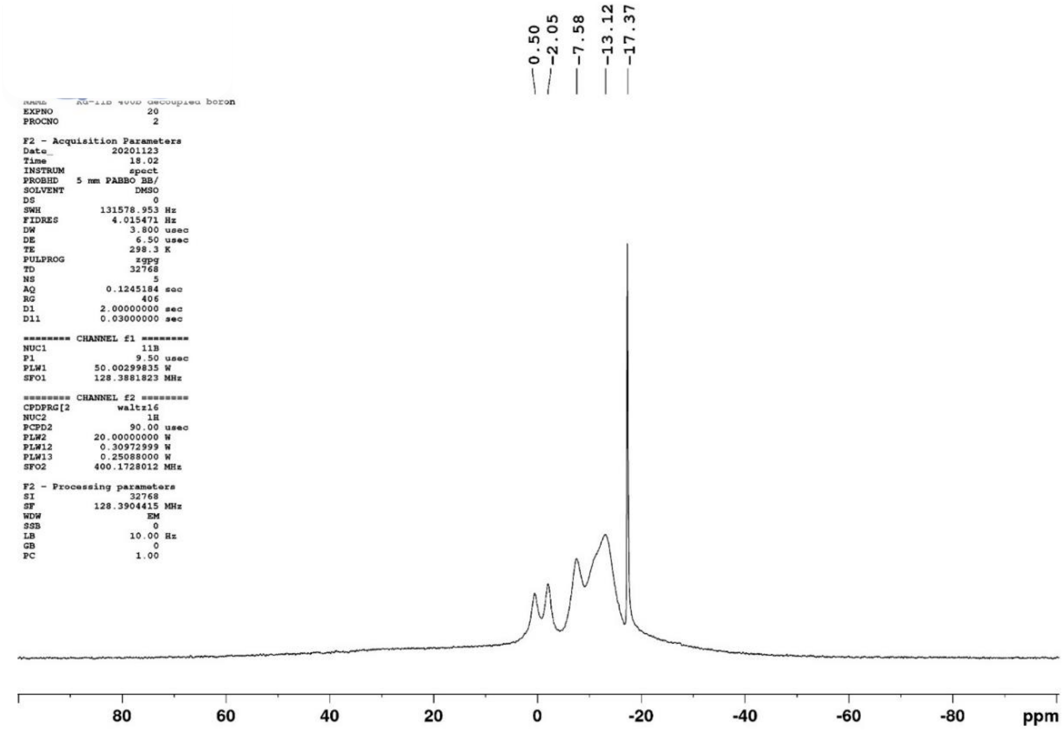

Figure S19. ^{19}F NMR of **3b**

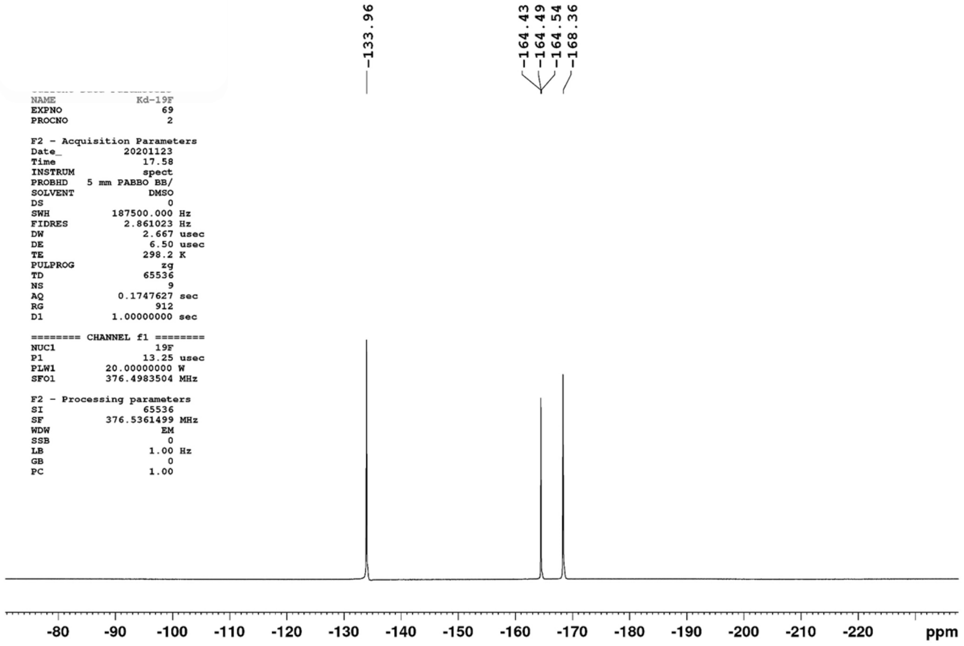

Figure S20. ^{31}P NMR: Reaction of **1** with **8**

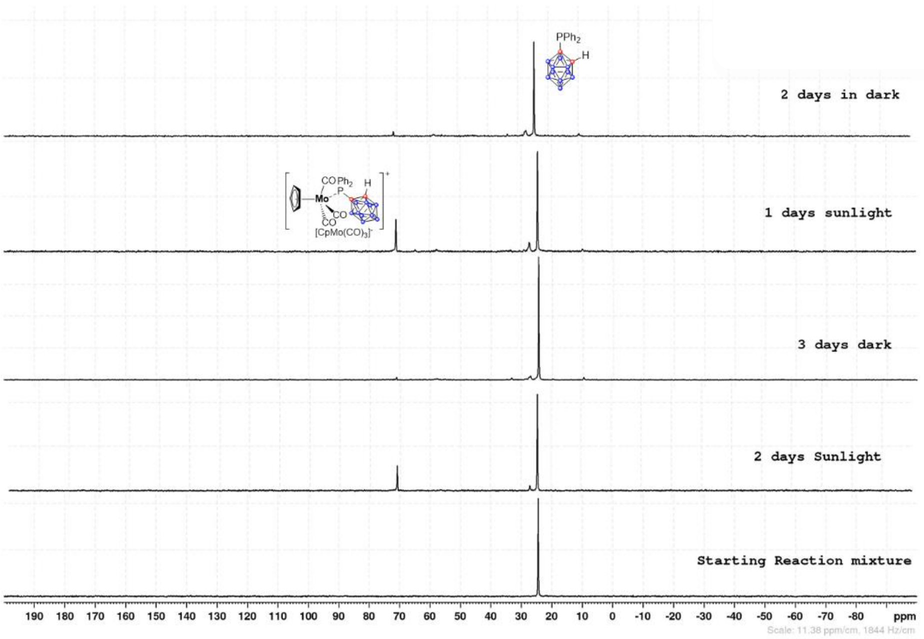

Figure S21. ^{31}P NMR: Reaction of **1** with PPh_3

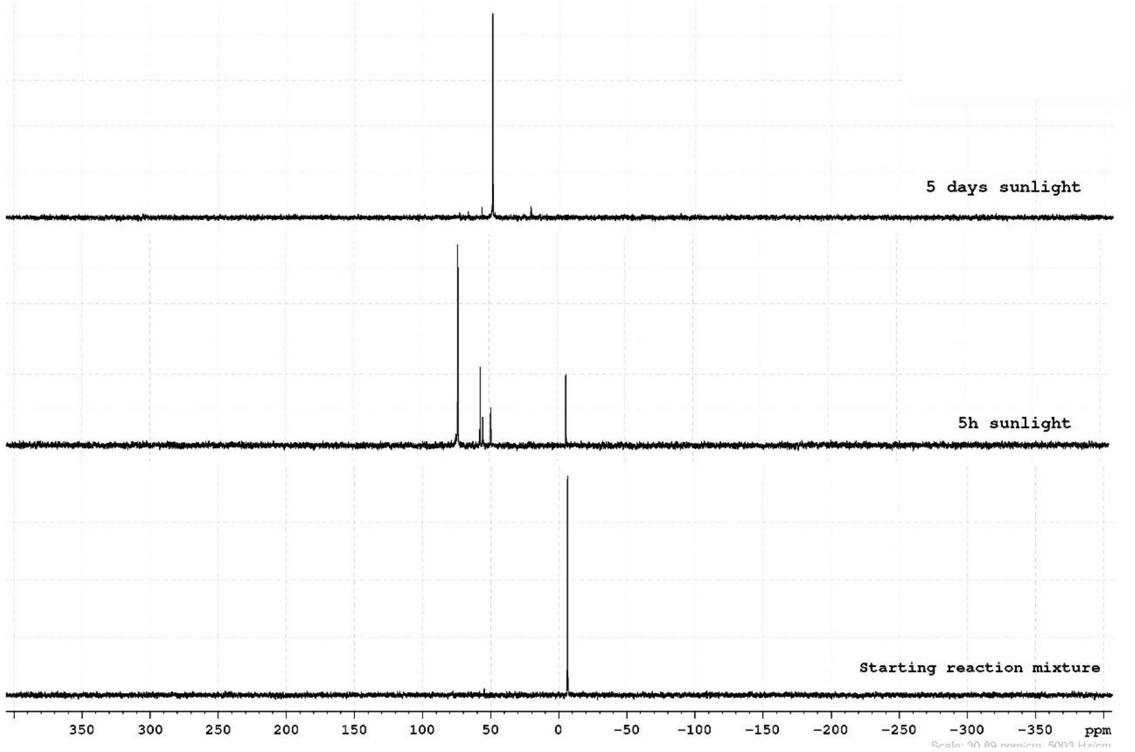

Figure S22. ^{31}P NMR: Reaction of **3b** with Cp^*_2Co after 10 min.

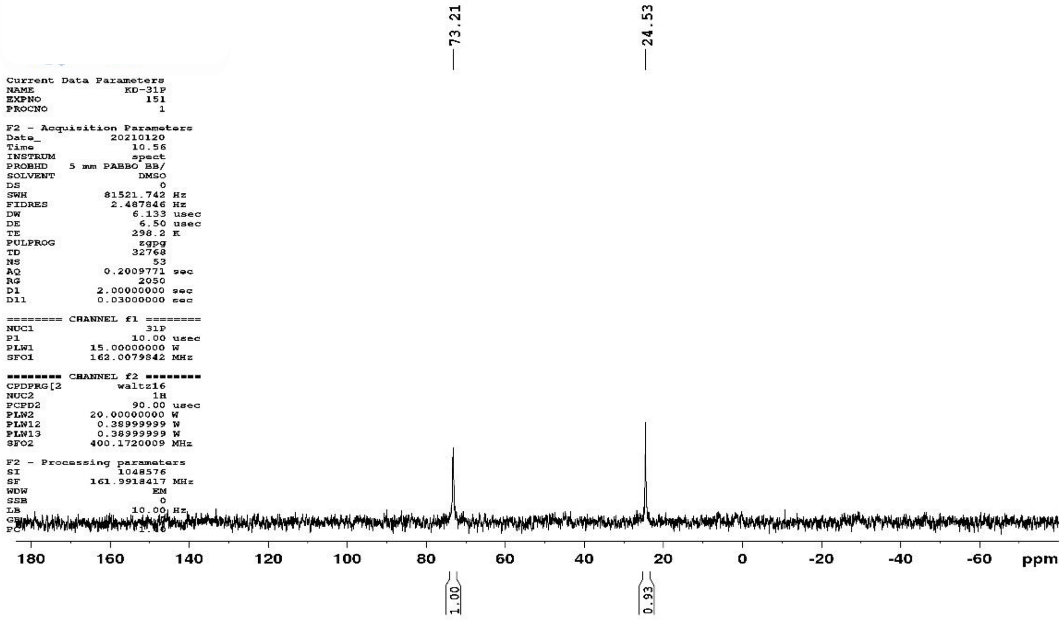

Figure S23. POV-ray structure of a new polymorph of [$Cp*_2Co^{III}$][$B(C_6F_5)_4$], thermal ellipsoids at the 50% probability level, hydrogens were omitted for clarity.[4]

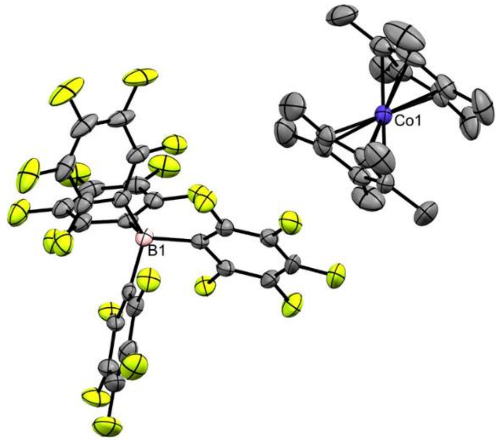

Figure S24.) EPR spectra of the reaction of **5b** (spectra of **5b** is given in red dotted line) with an exess of Ph_3CCl in pentane solution recorded after 10 min. - generation of $Ph_3C^{\bullet}$ (blue).

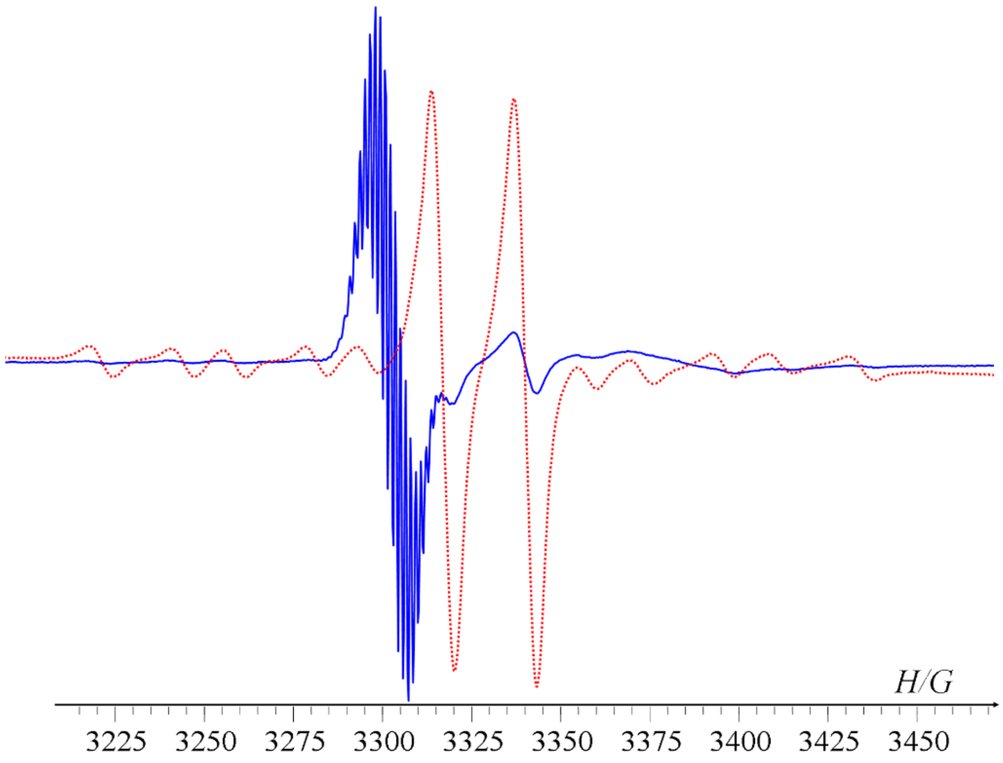

Figure S25. a) EPR spectra recorded over the course of the reaction of C_{60}, $[Cp(CO)_3Mo]_2$ (**1**) and PPh_3 in a molar ratio of 1:1:10 carried out in toluene under UV-irradiation after 10 min. from the beginning (top), and after 20 min. from the beginning (bottom); b) APCI MS in the positive mode of the reaction mixture of C_{60}, **1** and PPh_3 in a molar ratio of 1:1:10 carried out in toluene under UV-irradiation in toluene solution after 10 min. (top), and simulated MS of the $C_{60}Ph(PPh_2)+H^+$ (bottom); c) APCI MS in the positive mode of the reaction mixture of C_{60}, **1** and PPh_3 in a molar ratio of 1:1:10 carried out in toluene under UV-irradiation in toluene solution after 20 min. (top), and simulated MS of the $C_{60}Ph_7+H^+$ (bottom).

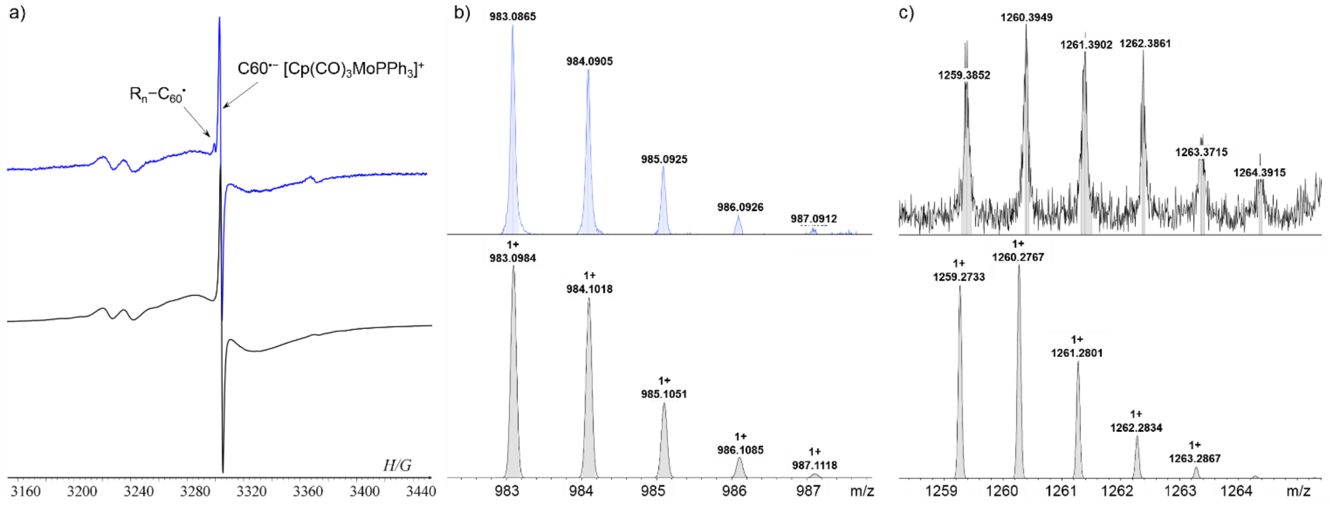

CV experiment of compound (3b)

The electrochemical phenomenon of the complex **3b** was studied using 5.99 mM solution of **3b** in a CH_2Cl_2 solution of $[nBu_4N][B(C_6F_5)_4]$ (0.1 M) as supporting electrolyte. The glassy carbon, Pt wire, and Ag wire were used as the working, counter and reference electrodes, respectively (Figure S26). The cyclic voltammograms (CVs) of the complex solution showed that, it can be reduced/oxidized upon alternation of voltage (-0 V to -2.2 V) results a well-defined reduction events at $E_{p,c-1}$ = -0.736 V and $E_{p,c-2}$ = -1.377, and well-defined oxidation events at $E_{p,a}$ = -0.341 V with peak to peak separation (ΔE) of 395 mV for $E_{p,a}$ *vs* $E_{p,c-1}$ peaks and 1036 mV for $E_{p,a}$ *vs* $E_{p,c-2}$ peaks at a scan rate of 100 mV/s (Figure S26). An exponential and linear dependence (R^2 = 0.99) of the peak currents versus scan rate and square root of these scan rates (100-900 mVs^{-1}) were observed (Figure S26), indicating a process controlled by diffusion.

Figure S26. (A) Cyclic voltammograms (CVs) of 5.99 mM solution of complex (**3b**) with scan rates of 100 mV/s using 0.1M $[nBu_4N][B(C_6F_5)_4]$ electrolyte in dry CH_2Cl_2. (B) Exponential and linear correlations between the peak currents ($E_{p,a}$ vs $E_{p,c-1}$) and scan rates (*v*) (left), and peak currents ($E_{p,a}$ vs $E_{p,c-1}$) and the square root of the scan rate ($v^{1/2}$) (right), respectively, during oxidation (top) and reduction (bottom) (R^2 > 0.99 for all fits). (C) Exponential and linear correlations between the peak currents ($E_{p,a}$ vs $E_{p,c-2}$) and scan rates (*v*) (left), and peak currents ($E_{p,a}$ vs $E_{p,c-2}$) and the square root of the scan rate ($v^{1/2}$) (right), respectively, during oxidation (top) and reduction (bottom) (R^2 > 0.99 for all fits)

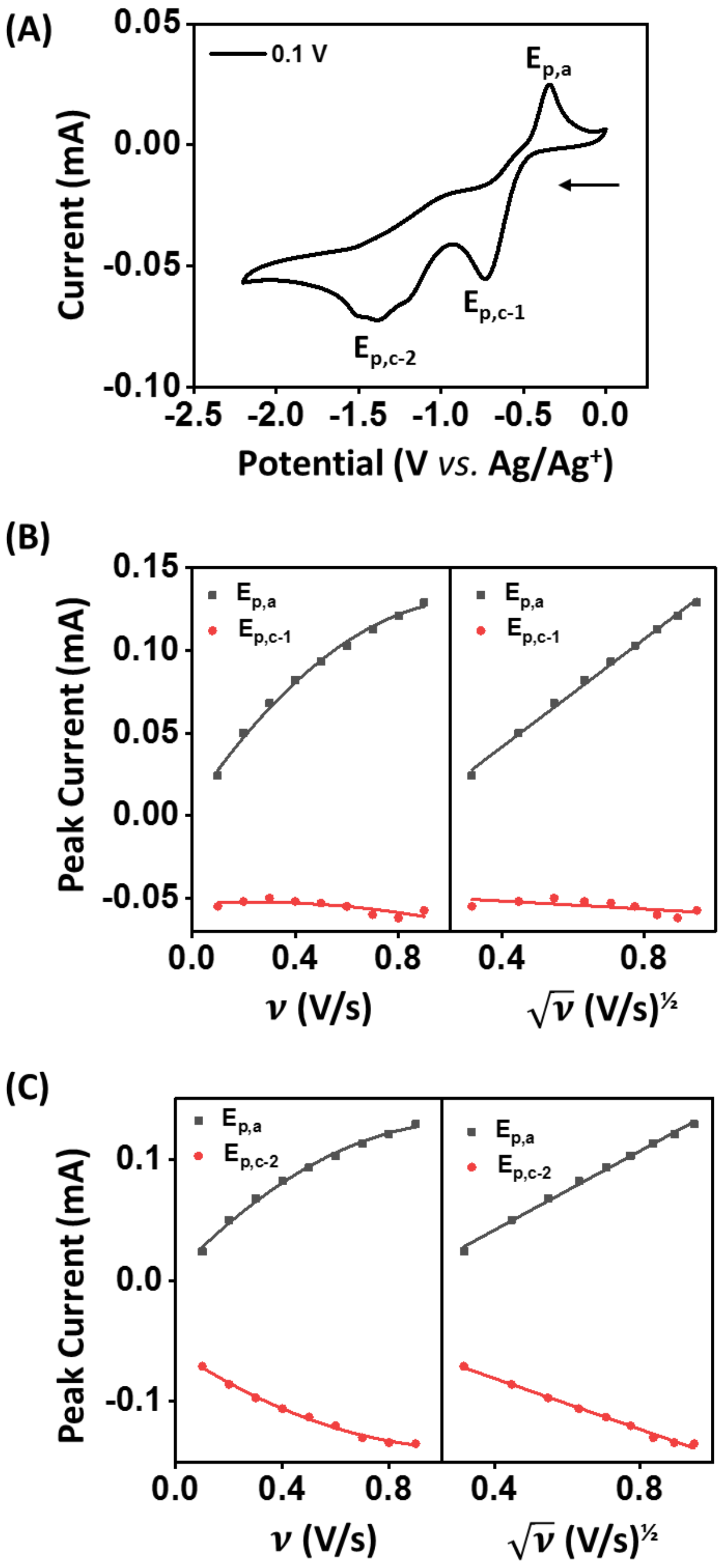

(A)
0.1 V
Ep,a
Ep,c-1
Ep,c-2
Current (mA)
Potential (V vs. Ag/Ag+)
(B)
Ep,a
Ep,c-1
Peak Current (mA)
ν (V/s)
√ν (V/s)½
(C)
Ep,a
Ep,c-2
Peak Current (mA)
ν (V/s)
√ν (V/s)½

CV experiment of compound (5b)

The electrochemical phenomenon of the radical **5b** was measured in a CH_2Cl_2 solution of $[nBu_4N][B(C_6F_5)_4]$ (0.1 M) as supporting electrolyte. The glassy carbon, Pt wire, and Ag wire were used as the working, counter and reference electrodes, respectively (Figure S27). The cyclic voltammograms (CVs) of the complex solution showed that, it can be reduced/oxidized upon alternation of voltage (-0 V to -2 V) results a well-defined reduction events at $E_{p,c1}$ = -1.236 V, and oxidation events at $E_{p,a}$ = -0.358 V with peak to peak separation (ΔE) of 878 mV at a scan rate of 100 mV/s (Figure S27).

Figure S27. Cyclic voltammograms (CVs) of 6.1 mM solution of complex (**5b**) with scan rates of 100 mV/s using 0.1M $[nBu_4N][B(C_6F_5)_4]$ electrolyte in dry CH_2Cl_2.

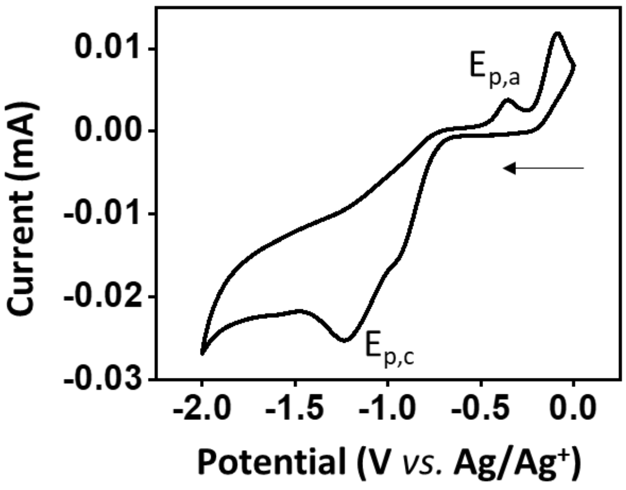

Figure S28. UV-Vis spectra of compound (5b)

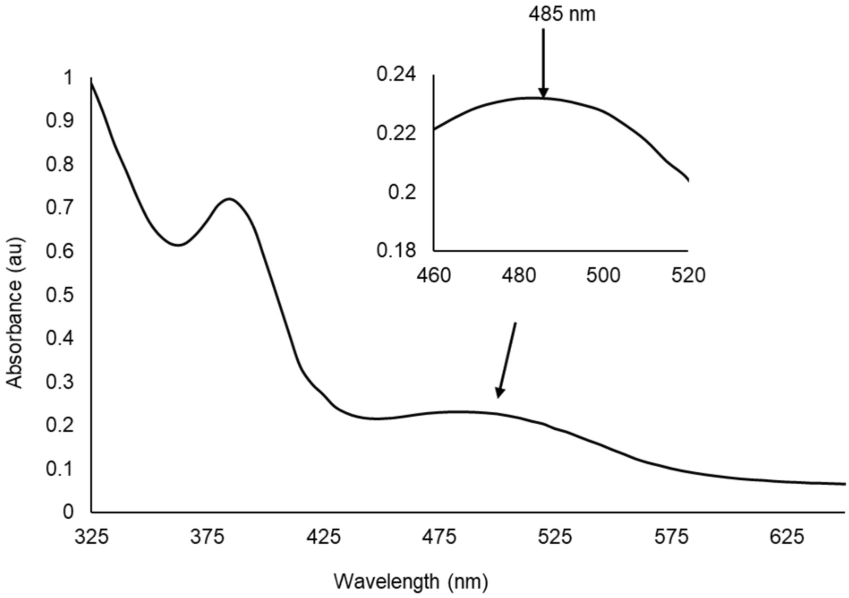

Figure S29. IR spectra of compound (5b)

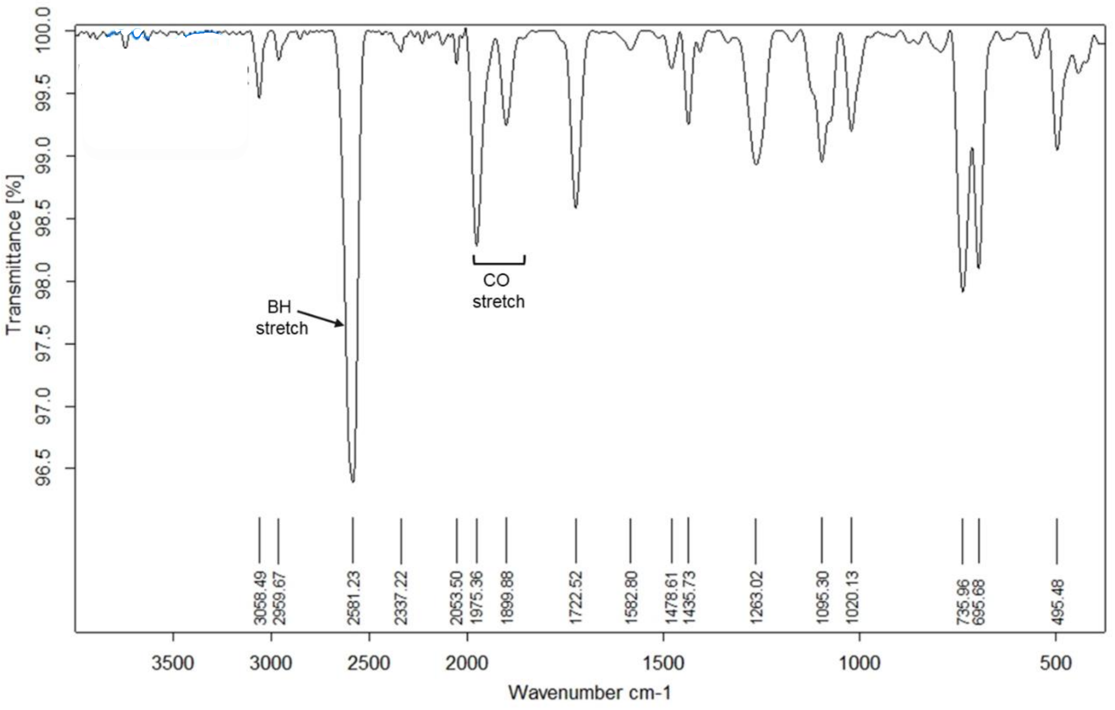

Figure S30. IR spectra of compound (3b)

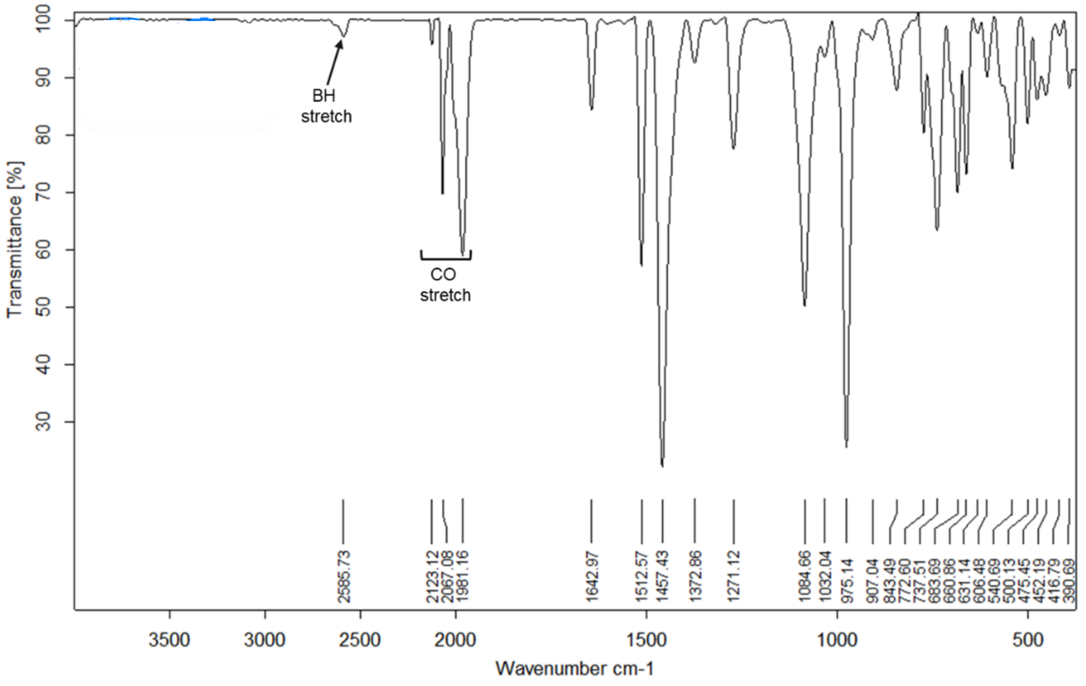

DFT Computations

DFT calculations were performed using Gaussian 09.2.[5] Geometry optimization of all the molecules were carried out using the uwB97XD method[6] with Ahlrichs' def2-SVP basis set,[7] and with the relativistic effect of molybdenum, which was accounted for by the Stuttgart-Dresden ECP,[8] implemented in the Gaussian 09 software. Thermal energy corrections were extracted from the results of frequency analysis performed at the same level of theory. Frequency analysis of all the molecules and intermediates contained no imaginary frequency showing that these are energy minima.

Optimized geometries

17-electron

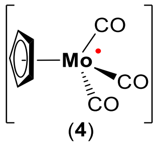

(**4**)

Mo	-0.05081	-0.00973	-0.13177
C	-1.20365	-1.53837	-0.72812
C	-1.24899	0.03619	1.43640
C	-1.19168	1.49770	-0.78391
O	-1.84073	-2.43456	-1.05846
O	-1.92220	0.06638	2.36856
O	-1.81963	2.38963	-1.14315
C	1.80363	-0.56739	1.18729
H	1.70076	-1.08178	2.14077
C	2.01595	-1.18398	-0.07431
C	1.80560	0.85089	0.99297
H	2.08473	-2.25435	-0.26004
C	2.15058	-0.15840	-1.04992
H	1.71545	1.60474	1.77220

C	2.01356	1.09708	-0.39307
H	2.33023	-0.30871	-2.11338
H	2.09343	2.07482	-0.86470

Sum of electronic and zero-point Energies= -601.131903

Sum of electronic and thermal Energies= -601.120288

Sum of electronic and thermal Enthalpies= -601.119344

Sum of electronic and thermal Free Energies= -601.171751

19-electron

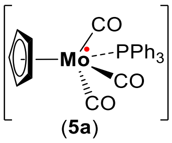

(**5a**)

Mo	-1.79962	0.32842	-0.09972
C	-1.40794	0.51023	-2.11429
C	-2.15872	2.19714	-0.51289
C	-1.43738	1.28825	1.62151
O	-1.08916	0.89883	-3.15382
O	-2.39165	3.30724	-0.74478
O	-1.16134	1.98442	2.50354
P	0.63257	-0.02874	0.00667
C	1.44203	-0.67557	-1.51323
C	0.80224	-1.70311	-2.21983
C	2.66817	-0.19196	-1.97813
C	1.38466	-2.24758	-3.35982
H	-0.16574	-2.07567	-1.87540
C	3.24445	-0.72934	-3.12980
H	3.17812	0.61362	-1.44655
C	2.60808	-1.75851	-3.81914

H	0.87623	-3.04975	-3.89874
H	4.19740	-0.33565	-3.48961
H	3.06144	-2.17700	-4.72018
C	1.07768	-1.31179	1.25916
C	0.67966	-1.11936	2.59060
C	1.77600	-2.47783	0.92785
C	0.96557	-2.07336	3.56217
H	0.15163	-0.20989	2.88043
C	2.05221	-3.43914	1.90206
H	2.11210	-2.64644	-0.09621
C	1.64725	-3.24177	3.21887
H	0.65323	-1.90233	4.59448
H	2.59298	-4.34638	1.62419
H	1.86536	-3.99424	3.97961
C	1.68436	1.40020	0.46746
C	2.80539	1.26963	1.29487
C	1.36541	2.65525	-0.06383
C	3.59112	2.38253	1.59107
H	3.06660	0.29712	1.71740
C	2.15805	3.76298	0.22549
H	0.48601	2.76712	-0.70138
C	3.26985	3.62914	1.05657
H	4.45805	2.27339	2.24612
H	1.89806	4.73683	-0.19381
H	3.88409	4.50068	1.29336
C	-3.94674	-0.73751	-0.49774
H	-4.69049	-0.24840	-1.12369
C	-3.04400	-1.75085	-0.91148
C	-3.77804	-0.54734	0.90569

H	-2.91285	-2.10542	-1.93239
C	-2.25566	-2.12181	0.20474
H	-4.37906	0.10185	1.53890
C	-2.73991	-1.41848	1.33876
H	-1.45982	-2.86570	0.21737
H	-2.36333	-1.52079	2.35456

Sum of electronic and zero-point Energies= -1636.332109

Sum of electronic and thermal Energies= -1636.303122

Sum of electronic and thermal Enthalpies= -1636.302178

Sum of electronic and thermal Free Energies= -1636.395300

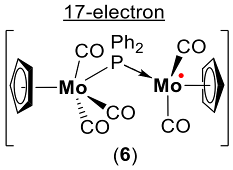

(**6**)

Mo	2.35458	-0.41410	-0.09273
C	2.48689	1.23953	1.01707
C	3.87874	0.54463	-0.92359
C	1.70314	-0.10329	-1.95878
O	2.64480	2.14901	1.69667
O	4.77528	1.07822	-1.39985
O	1.39977	0.00987	-3.05537
P	-0.13065	0.44120	0.14386
C	-0.50429	0.97205	1.87771
C	0.07903	0.34874	2.98573
C	-1.50463	1.92997	2.10730
C	-0.32222	0.66517	4.28301
H	0.86438	-0.39254	2.84108

C	-1.90095	2.25360	3.40166
H	-1.98070	2.43033	1.26165
C	-1.31337	1.61957	4.49663
H	0.14849	0.16398	5.13153
H	-2.67609	3.00785	3.55508
H	-1.62528	1.87127	5.51235
C	-0.23733	2.03670	-0.78873
C	-0.76976	2.02190	-2.08436
C	0.21586	3.25731	-0.26833
C	-0.83852	3.18907	-2.84380
H	-1.14043	1.08271	-2.50249
C	0.13934	4.42548	-1.02320
H	0.61913	3.30886	0.74411
C	-0.38479	4.39555	-2.31543
H	-1.25508	3.15175	-3.85263
H	0.49322	5.36607	-0.59542
H	-0.44264	5.31238	-2.90603
C	3.89282	-1.94687	0.73781
H	4.94272	-1.69355	0.87337
C	2.87914	-1.84599	1.72797
C	3.30208	-2.48802	-0.44551
H	3.01905	-1.48945	2.74720
C	1.66646	-2.32255	1.16436
H	3.82356	-2.73055	-1.36926
C	1.91898	-2.71330	-0.17879
H	0.70839	-2.40414	1.67125
H	1.19265	-3.14037	-0.86652
Mo	-2.09430	-1.03317	-0.38497
C	-1.63801	-2.60237	0.66891

C	-1.11386	-2.02065	-1.74009
O	-1.42585	-3.57007	1.27855
O	-0.54459	-2.64572	-2.53929
C	-3.62026	0.77125	-0.71287
H	-3.29221	1.80512	-0.80745
C	-3.76661	-0.15362	-1.78379
C	-4.01196	0.12095	0.49010
H	-3.59163	0.05242	-2.83813
C	-4.24133	-1.38654	-1.23872
H	-4.01294	0.56051	1.48624
C	-4.38371	-1.21129	0.16513
H	-4.49027	-2.28468	-1.79974
H	-4.73695	-1.96270	0.86910

Sum of electronic and zero-point Energies= -1892.995297

Sum of electronic and thermal Energies= -1892.961496

Sum of electronic and thermal Enthalpies= -1892.960552

Sum of electronic and thermal Free Energies= -1893.064055

19-electron

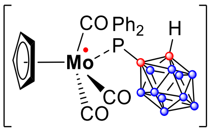

(**5b**)

Mo	-1.95075	-0.60919	-0.43659
C	-0.96483	-1.30364	-2.07572
C	-2.77070	0.36595	-1.90432
C	-2.57509	1.11549	0.44485
O	-0.41267	-1.41859	-3.08871

O	-3.30231	0.91099	-2.77514
O	-2.88360	2.18033	0.75554
P	0.21297	0.49917	0.10062
C	0.13517	1.15927	1.82689
C	0.17681	2.52617	2.12631
C	-0.18585	0.26691	2.85961
C	-0.02837	2.97553	3.43074
H	0.33559	3.26667	1.34479
C	-0.37702	0.71217	4.16261
H	-0.31022	-0.79074	2.64254
C	-0.28848	2.07217	4.45586
H	0.00732	4.04697	3.63693
H	-0.61022	-0.00864	4.94875
H	-0.44431	2.42722	5.47655
C	0.71719	1.93764	-0.92293
C	1.73670	2.81795	-0.52174
C	0.08868	2.15341	-2.15130
C	2.09415	3.89691	-1.32011
H	2.27188	2.65037	0.41376
C	0.45701	3.22972	-2.95987
H	-0.69781	1.47857	-2.48307
C	1.45313	4.10608	-2.54307
H	2.88564	4.57309	-0.99149
H	-0.04641	3.37884	-3.91669
H	1.73758	4.95249	-3.17184
C	-3.43036	-2.52627	-0.42999
H	-3.98795	-2.77582	-1.33050
C	-2.20591	-3.11056	-0.01724
C	-3.85109	-1.62735	0.59358

H	-1.61661	-3.82434	-0.58965
C	-1.82587	-2.52105	1.21194
H	-4.79062	-1.07884	0.61430
C	-2.86430	-1.64181	1.61744
H	-0.92933	-2.76914	1.77865
H	-2.88933	-1.06030	2.53734
C	1.82895	-0.56211	0.11490
B	3.24392	0.03158	-0.71510
B	1.61705	-2.27271	-0.08867
B	2.22050	-1.59000	1.42881
B	3.20551	-0.15925	1.05051
C	2.31181	-1.27495	-1.27966
H	3.19638	1.05029	-1.31769
B	4.61553	-0.71131	0.10343
B	4.00437	-1.39725	-1.42285
H	0.51811	-2.68222	-0.26388
B	3.00854	-2.81210	-1.04386
B	2.99483	-3.02209	0.72123
H	1.52040	-1.61227	2.39025
B	3.98898	-1.71785	1.43166
H	3.14085	0.81550	1.74269
H	1.70091	-1.07515	-2.15902
H	5.65744	-0.12025	0.14985
B	4.48272	-2.48229	-0.10011
H	4.48092	-1.26830	-2.51203
H	2.80871	-3.64930	-1.87389
H	2.85724	-4.10017	1.22773
H	4.58090	-1.86217	2.46455
H	5.44838	-3.18543	-0.20297

Sum of electronic and zero-point Energies= -1736.039647

Sum of electronic and thermal Energies= -1736.007912

Sum of electronic and thermal Enthalpies= -1736.006968

Sum of electronic and thermal Free Energies= -1736.101887

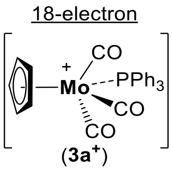

(3a+)

Mo	1.85005	0.14184	0.09350
C	1.27644	1.00220	1.83245
C	3.04121	1.76147	0.22152
C	1.41338	1.16974	-1.57683
O	1.01172	1.46649	2.83927
O	3.72752	2.66820	0.28822
O	1.23974	1.71938	-2.56191
P	-0.70065	-0.01372	-0.00903
C	-1.52149	-0.38090	1.58220
C	-0.94759	-1.29548	2.47643
C	-2.74441	0.21807	1.90638
C	-1.59045	-1.61338	3.66895
H	0.01069	-1.76401	2.24740
C	-3.38133	-0.09729	3.10553
H	-3.20566	0.93956	1.23006
C	-2.80898	-1.01248	3.98568
H	-1.13592	-2.32771	4.35789
H	-4.33157	0.38005	3.35112
H	-3.30949	-1.25496	4.92497

C	-1.20888	-1.36381	-1.13373
C	-0.87511	-1.27834	-2.49393
C	-1.90181	-2.48501	-0.66537
C	-1.22577	-2.30260	-3.36755
H	-0.36147	-0.39850	-2.88742
C	-2.24139	-3.51476	-1.54470
H	-2.18713	-2.56168	0.38559
C	-1.90359	-3.42717	-2.89247
H	-0.97510	-2.21936	-4.42678
H	-2.78114	-4.38624	-1.16972
H	-2.17606	-4.23093	-3.57898
C	-1.54430	1.47725	-0.63362
C	-2.68463	1.37021	-1.43999
C	-1.09560	2.74317	-0.23552
C	-3.36059	2.51934	-1.84616
H	-3.05197	0.39095	-1.75374
C	-1.77804	3.88698	-0.63896
H	-0.20377	2.84389	0.38650
C	-2.90983	3.77620	-1.44648
H	-4.24564	2.42954	-2.47864
H	-1.42056	4.86930	-0.32511
H	-3.44069	4.67439	-1.76766
C	3.70810	-1.13624	0.62468
H	4.55471	-0.74007	1.18309
C	2.59817	-1.82989	1.17922
C	3.55358	-1.11602	-0.79886
H	2.44204	-2.03740	2.23672
C	1.75472	-2.23048	0.11004
H	4.26777	-0.71306	-1.51479

C	2.33734	-1.78935	-1.11278
H	0.83290	-2.80447	0.19350
H	1.93904	-1.98175	-2.10717

Sum of electronic and zero-point Energies= -1636.174906

Sum of electronic and thermal Energies= -1636.146748

Sum of electronic and thermal Enthalpies= -1636.145804

Sum of electronic and thermal Free Energies= -1636.235703

18-electron

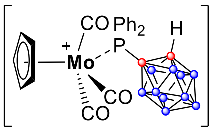

($\mathbf{3b^+}$)

Mo	-1.91735	-0.88292	-0.16720
C	-0.84649	-1.86005	-1.56995
C	-3.23629	-1.23877	-1.65701
C	-2.50311	0.94505	-0.76937
O	-0.30649	-2.46506	-2.37448
O	-3.98526	-1.43894	-2.48983
O	-2.92939	1.95412	-1.08770
P	0.25042	0.51104	0.04536
C	0.02997	1.64801	1.47122
C	-0.73018	2.80780	1.24060
C	0.43172	1.35922	2.78068
C	-1.07906	3.64724	2.29410
H	-1.04591	3.08146	0.23382
C	0.08343	2.20637	3.83170
H	1.03112	0.48118	3.00308

C	-0.67662	3.34843	3.59504
H	-1.66265	4.54687	2.09055
H	0.41789	1.96743	4.84278
H	-0.94821	4.00995	4.41968
C	0.56096	1.61623	-1.37202
C	1.25486	2.82245	-1.18606
C	0.16836	1.24381	-2.66246
C	1.53894	3.63957	-2.27362
H	1.58763	3.12217	-0.19060
C	0.46984	2.05853	-3.75271
H	-0.39068	0.32246	-2.82977
C	1.15087	3.25757	-3.55883
H	2.07355	4.57807	-2.11787
H	0.16023	1.75717	-4.75486
H	1.37993	3.89974	-4.41138
C	-3.16685	-2.45525	0.99724
H	-3.80401	-3.18923	0.50568
C	-1.82138	-2.66397	1.39924
C	-3.56387	-1.14364	1.41819
H	-1.24012	-3.57231	1.25051
C	-1.37756	-1.48601	2.04926
H	-4.55601	-0.70949	1.30688
C	-2.44957	-0.53917	2.06300
H	-0.39688	-1.34962	2.49841
H	-2.42306	0.44433	2.52980
C	1.90569	-0.40525	0.27125
B	3.28120	0.23172	-0.59649
B	1.85118	-2.14321	0.25934
B	2.35787	-1.24972	1.69616

B	3.22591	0.21986	1.17862
C	2.47394	-1.21423	-1.03237
H	3.14029	1.18572	-1.28536
B	4.69258	-0.30344	0.30891
B	4.17289	-1.19638	-1.14495
H	0.79249	-2.66881	0.14815
B	3.29805	-2.65122	-0.62603
B	3.27064	-2.67363	1.15458
H	1.62539	-1.25955	2.63711
B	4.13011	-1.21614	1.73293
H	3.06751	1.26023	1.74486
H	1.87096	-1.15639	-1.93848
H	5.67217	0.38331	0.30337
B	4.71814	-2.08973	0.29327
H	4.65197	-1.13825	-2.23728
H	3.18086	-3.58249	-1.36463
H	3.21016	-3.69839	1.77032
H	4.70681	-1.19448	2.78167
H	5.74153	-2.70910	0.27707

Sum of electronic and zero-point Energies= -1735.869790

Sum of electronic and thermal Energies= -1735.838811

Sum of electronic and thermal Enthalpies= -1735.837867

Sum of electronic and thermal Free Energies= -1735.929664

Scheme S2. Comparison in stability of 5a vs. 3b in one e reduction reaction.

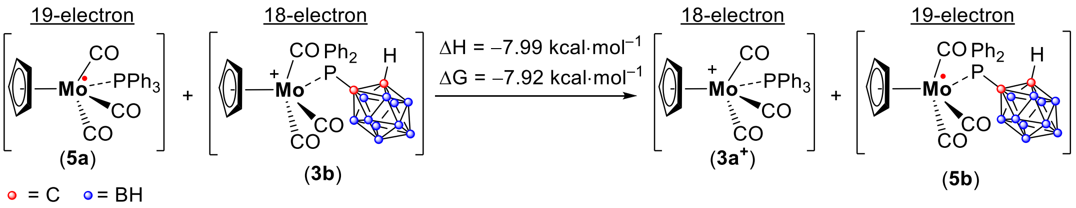

Figure S27. TD-DFT calculations of **5a** and its analysis.

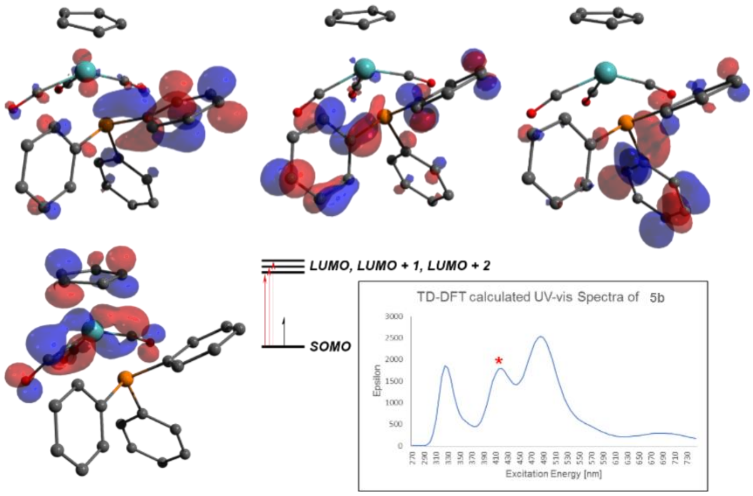

EPR calculations of radicals 4, 6 and 5b.

The EPR calculations for radicals **4**, **6** and **5b** were carried out using the ORCA 4.0 software[9] at unrestricted TPSS0/def2-TZVP level of theory[10] with the relativistic effect, which was accounted for by the zeroth-order regular approximation (ZORA).[11] The calculated EPR parameters and spin density distributions are summarized in Tables 1 and 2, respectively.

Table S1. Experimental and DFT calculated EPR parameters for radicals **4**, **6** and **5b**.

	g_{iso}	$a(^{95,97}Mo)$ [G]	$a(^{31}P)$ [G]
**4** (Experimental)	2.082	-	-
**4** (Calculated)	2.069	13.3	-
**6** (Experimental)	2.044	12.4	14.2
**6** (Calculated)	2.053	20.9	30.5
**5b** (Experimental)	1.980	36.0	21.3
**5b** (Calculated)	1.990	23.4	10.8

Table S2. Calculated Mulliken spin density of radicals **4**, **6** and **5b**.

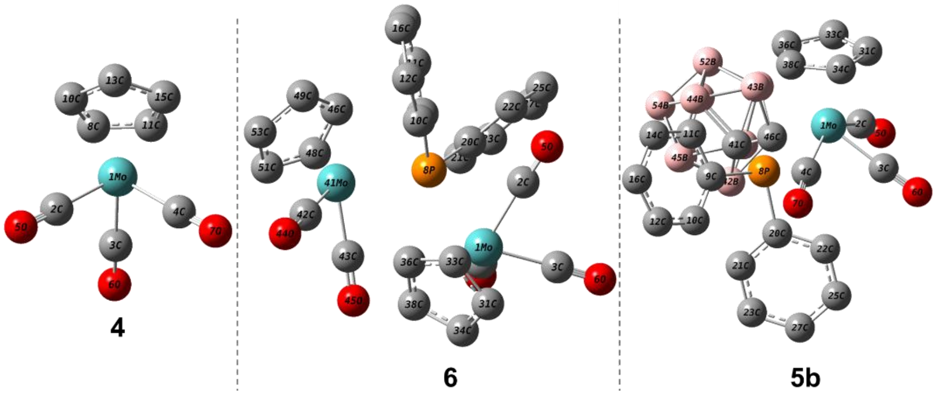

4 **6** **5b**

	1Mo	13C	41Mo	49C	2C	4C	33C	38C
**4**	0.80	0.10	-	-	-	-	-	-
**6**	-	-	0.85	0.10	-	-	-	-
**5b**	0.22	-	-	-	0.18	0.14	0.16	0.15

References

[1] C. Viñas, R. Benakki, F. Teixidor, J. Casabó, *Inorg. Chem*. **1995**, *34*, 3844-3845; b) S. A. Keppie, M. F. Lappert, *J. Chem. Soc. A*, **1971**, 3216-3220; c) D. H. Gibson, K. Owens, S. K. Mandal, W. E. Sattich, J. 0. Franco, *Organometallics* **1989**, *8*, 498-505.

[2] a) B. Tumanskii, O. Kalina, Radical Reactions of Fullerenes and Their Derivatives. Developments in Fullerene Studies, 2, Kluwer Academic Publishers, Dordrecht, the Netherlands, **2001**; b) M. D. Tzirakis, M. Orfanopoulos, *Chem. Rev*. **2013**,*113*, 5262-5321.

[3] M. A. Greaney, S. M. Gorun, *J. Phys. Chem*. **1991**, *95*, 7142-7144.

[4] L.L.Cao, D.W.Stephan, *Organometallics* **2017,** *36*, 3163-3170.

[5] Gaussian 09, Revision D.01, M. J. Frisch, G. W. Trucks, H. B. Schlegel, G. E. Scuseria, M. A. Robb, J. R. Cheeseman, G. Scalmani, V. Barone, B. Mennucci, G. A. Petersson, H. Nakatsuji, M. Caricato, X. Li, H. P. Hratchian, A. F. Izmaylov, J. Bloino, G. Zheng, J. L. Sonnenberg, M. Hada, M. Ehara, K. Toyota, R. Fukuda, J. Hasegawa, M. Ishida, T. Nakajima, Y. Honda, O. Kitao, H. Nakai, T. Vreven,

J. A. Montgomery, Jr., J. E. Peralta, F. Ogliaro, M. Bearpark, J. J. Heyd, E. Brothers, K. N. Kudin, V. N. Staroverov, T. Keith, R. Kobayashi, J. Normand, K. Raghavachari, A. Rendell, J. C. Burant, S. S. Iyengar, J. Tomasi, M. Cossi, N. Rega, J. M. Millam, M. Klene, J. E. Knox, J. B. Cross, V. Bakken, C. Adamo, J. Jaramillo, R. Gomperts, R. E. Stratmann, O. Yazyev, A. J. Austin, R. Cammi, C. Pomelli, J. W. Ochterski, R. L. Martin, K. Morokuma, V. G. Zakrzewski, G. A. Voth, P. Salvador, J. J. Dannenberg, S. Dapprich, A. D. Daniels, O. Farkas, J. B. Foresman, J. V. Ortiz, J. Cioslowski, D. J. Fox, *Gaussian*, Inc., Wallingford CT, **2013**.

[6] a) F. Weigend, R. Ahlrichs, *Phys. Chem. Chem. Phys.* **2005**, *7*, 3297-3305; b) J.-D. Chai, M. Head-Gordon, *J. Chem. Phys.* **2008**, *128*, 084106; c) J.-D. Chai, M. Head-Gordon, *Phys. Chem. Chem. Phys.* **2008**, *10*, 6615-6620; c) S. Grimme, *J. Chem. Phys. A* **2005**, *109*, 3067-3077; d) M. M. Quintal, A. Karton, M. A. Iron, A. D. Boese, J. M. L. Martin, *J. Chem. Phys. A* **2006**, *110*, 709-716.

[7] P. Schwerdtfeger, M. Dolg, W. H. E. Schwarz, G. A. Bowmaker, P. D. W. Boyd, *J. Chem. Phys.* **1989**, *91*, 1762-1774.

[8] a) U. Wedig, M. Dolg, H. Stoll, and H. Preuss, *in Quantum Chemistry: The Challenge of Transition Metals and Coordination Chemistry*, ed. A. Veillard **1986,** *79*; b) D. Andrae, U. Haeussermann, M. Dolg, H. Stoll, and H. Preuss, *Theor. Chem. Acc*. **1990**, *77,* 123-141.

[9] The calculations were carried out using F. Neese, *ORCA*, version 4.0, an ab initio, density functional and semi-empirical program package (Max Planck Institute for Bioinorganic Chemistry: Mulheim an der Ruhr, Germany, **2012**).

[10] a) D. A. Pantazis, X.-Y. Chen, C. R. Landis, F. Neese, *J. Chem. Theory Comput.* **2008**, *4*, 908-919; b) A. Schäfer, C. Huber, R. Ahlrichs, *J. Chem. Phys.* **1994**, *100*, 5829-5835; c) A. Schäfer, H. Horn, R. Ahlrichs, *J. Chem. Phys.* **1992**, *97*, 2571-2577.

[11] a) E. v. Lenthe, E. J. Baerends, J. G. Snijders, *J. Chem. Phys.* **1993**, *99*, 4597-4610; b) E. v. Lenthe, E. J. Baerends, J. G. Snijders, *J. Chem. Phys.* **1994**, *101*, 9783-9792; c) C. v. Wüllen, *J. Chem. Phys.* **1998**, *109*, 392-399.
